# A meta-analysis of behaviour change techniques in social interventions targeting improved cognitive function in older adults

**DOI:** 10.1186/s12889-025-22229-x

**Published:** 2025-03-27

**Authors:** Joyce Siette, Victoria Chong, Suraj Samtani, Celia B. Harris, Genevieve Z. Steiner-Lim, Freya MacMillan

**Affiliations:** 1 The MARCS Institute for Brain, Behaviour and Development, Western Sydney University, Locked Bag 1797, Penrith, New South Wales 2751 Australia; 2 Centre for Healthy Brain Ageing (CHeBA), Discipline of Psychiatry & Mental Health, School of Clinical Medicine, UNSW Sydney, Sydney, NSW 2052 Australia; 3 NICM Health Research Institute, Western Sydney University, Penrith, New South Wales 2751 Australia; 4 School of Health Sciences, Western Sydney University, Penrith, New South Wales 2751 Australia

**Keywords:** Behaviour change, Dementia, Cognition, Older adults, Social capital

## Abstract

**Background:**

Limited social networks in older adults are linked with increased dementia risk. However, there is a lack of knowledge on whether socially-based behavioural interventions (i.e., programs designed to increase individual’s social opportunities, engagement or networks) can improve cognitive function, as well as the role of applied behaviour change techniques (BCTs) in effective interventions. This systematic review and meta-analysis aimed to (i) quantify the effectiveness of social-based behavioural interventions in improving cognition in older adults, and (ii) identify which BCTs increase social activity behaviour of older adults.

**Methods:**

Six electronic databases were searched with restrictions for age (>65 years) and English language from inception to July 2023 (PROSPERO:CRD42021283382) for articles reporting social-based behavioural randomised controlled trials and using a measured outcome of cognitive function. Behaviour change techniques were mapped to the BCT V1 model and risk of bias was assessed. Pooled effect sizes from eligible studies were synthesised using RevMan.

**Results:**

We identified 9528 records and included 15 studies (*N*=1785 participants). Meta-analyses showed that social-based interventions had a medium effect on global cognition (*d*=0.80, 95% CI 0.58 to 1.02, *p*<0.01), but not executive function. The most frequently used intervention components were social-based communication (e.g., chatting, boosting social engagement), group arts-based tasks (e.g., knitting, music, craft) and guided reminiscence. The BCT demonstration of behaviour predicted significant cognitive effects and explained 94.6% of inter-study variation.

**Discussion:**

Findings carry implications for developing comprehensive strategies to promote social initiatives supporting cognitive health, particularly in addressing the challenges faced by older adults.

**Supplementary Information:**

The online version contains supplementary material available at 10.1186/s12889-025-22229-x.

## Background

Increase in life expectancy and population ageing is a global phenomenon [1]. An increasingly high number of older adults, especially women, are living alone [2]. About half of those 60 years and older are at risk of being socially isolated and approximately 30% of the oldest old (aged 85+ years) may experience some degree of loneliness [3–6]. Social isolation, particularly among older adults living alone, has been linked to poorer eating habits, challenges in instrumental activities of daily living, and a heightened vulnerability to a range of health issues, including recent illness and falls [7–9]. Additionally, individuals experiencing social isolation face an elevated risk of various adverse health outcomes, such as depression, cardiovascular diseases, diabetes, cardiovascular death, and non-cardiovascular death [10].

Albeit less widely recognised, social isolation among older adults is associated with an increased risk of dementia and cognitive decline [11–14], attributed to factors such as diminished cognitive stimulation, heightened loneliness and depression, chronic stress, inflammation, and impaired neuroplasticity [15, 16]. Consequently, addressing social isolation and enabling social engagement may present important strategies for mitigating dementia risk and maintaining cognition in older age. We aimed to examine evidence that intervening to enhance social engagement could improve cognition in older adults.

Social isolation and loneliness are distinct but interrelated concepts. While loneliness is the subjective feeling of being lonely, social isolation is typically defined as the objective lack or limited extent of social contact (e.g., varying due to marital status, living alone or with others) [17, 18]. The prevalence of social isolation varies across different studies. Recent evidence showed higher rates in middle and high-income countries and urban areas [10]. However, previous studies have shown prevalence values ranging from 20% to 34% in European countries [19], 31% in Japan [20], 24% in the USA [21], 14% in Australia [22], and 15% in Brazil [23].

The significant prevalence of social isolation among older adults, coupled with its documented adverse effects on health and wellbeing, emphasises the imperative to address social isolation as a public health concern. Despite the robust evidence supporting interventions targeted at mitigating social isolation and the implementation of strategies to enhance its impact (e.g., [24–30]), a substantial gap persists in understanding the characteristics and effectiveness of interventions aimed at improving cognition among these individuals. Therefore, elucidating the mechanisms by which social isolation influences cognitive function and exploring the potential efficacy of interventions targeting both social isolation and cognitive decline are necessary avenues for future research in gerontology and public health. However, bridging the gap between evidence-backed interventions and their effective implementation is compounded by the challenge of recruiting socially isolated individuals which necessitates thoughtful approaches to ensure the success of intervention strategies.

Empirical studies have identified preliminary efficacy of social-based behavioural interventions in ameliorating social isolation and loneliness across individual, community and societal dimensions [31–33]. These interventions often encompass methodologies or programs crafted to alter or influence individual behaviours within social environments or contexts [34–37]. For instance, social-based behavioural initiatives strategically use social interactions, norms, and networks to stimulate behavioural change, advocate healthier decision-making, or tackle targeted issues such as chronic health outcomes, environmental sustainability, or community engagement [38–40]. They frequently rely on mechanisms such as social support, peer influence, social norms, or community resources to facilitate constructive behavioural transformations and augment overall wellbeing [40, 41]. Individual-focused interventions, encompassing both face-to-face and digital modalities, include social skills training, peer support, social activity groups, befriending services, and cognitive-behavioural therapy [26, 42, 43]. Community-level interventions target enhancements in transportation, improvements to the built environment's accessibility, and digital inclusion [44, 45]. At the societal level, interventions concentrate on augmenting social cohesion and mitigating marginalisation [31].

Central to these interventions are active components strategically designed to induce behavioural change, which are often encapsulated within Michie et al.'s [46] hierarchical international taxonomy of 93 Behaviour Change Techniques (BCTs), facilitating consensus in reporting behavioural change interventions and contributing to enhanced clarity and standardisation in intervention research. Building on the established role of BCTs in addressing risk factors associated with chronic health conditions such as diabetes [47–49], BCT taxonomies have also been specifically tailored for modifying unhealthy habits, such as smoking [50] and alcohol consumption [51]. Despite the wealth of information on BCT applications in varied health contexts, a gap exists regarding the identification of BCTs employed to encourage older adults to enhance social engagement and the potential cognitive benefits that could be derived from such interventions.

Therefore, this review aimed to identify the effectiveness of social-based behavioural interventions on cognition in older individuals while offering a comprehensive synthesis of the BCTs embedded within these social programs.

## Methods

### Registration

We used the Preferred Reporting Items for Systematic Reviews and Meta-Analyses Protocol (PRISMA-P) [52] to guide reporting (see Additional file 1, Supplementary Table 1). This protocol was registered on the PROSPERO database (CRD42021283382) before the search commenced.

### Inclusion and exclusion criteria

Randomised controlled trials were eligible for inclusion if they involved reported use of a social program (intervention) compared to usual care, active/passive control or no intervention (comparator), and included at least one measure of cognition (outcomes). In cases where the age range was not explicitly stated, we took an inclusive approach and included studies where the mean age of participants was over 65 years. While there may have been studies with mixed age groups or only reported mean or median age, articles were included if the focus was on older adults to maintain consistency and relevance to our research question.

Due to the broad definition of “social” interventions, articles were included if the main component contained any type of social-based interventions focused on alleviating loneliness and/or targeted improvement in social behaviour. This may include but was not restricted to structured reminiscence individual or group therapy, social group gatherings/excursions, psychodynamic therapy, mindfulness therapy, cognitive-based interventions, videoconference program, peer support network, laughter therapy, broad public health campaigns (e.g., media campaigns), web and smartphone applications. That is, interventions were included that contained some element of contact and participation with other people (beyond the research team). Interventions were also considered social-based behavioural programs if they explicitly articulated social aims or incorporated components designed to modify social interactions, norms, or networks. This might therefore encompass interventions building social engagement, promoting peer support, facilitating social interactions, or targeting social norms.

Studies were also only included if cognitive performance was one of the measured outcomes. Studies were excluded if it included populations with a previous history or symptoms of dementia (e.g., Alzheimer's disease), or mild cognitive impairment. There were no restrictions on gender type, occupation, or living arrangements.

### Search strategy

A systematic search of six electronic databases MEDLINE, EMBASE, PsycINFO, PubMed, Ovid and CINAHL was conducted with the assistance of a trained librarian from inception to 9 August 2022, and an updated search was conducted on 7 July 2023 to capture any new publications from the original search timepoint for review finalisation. Search terms were based on a combination of descriptors including MeSH terms for social isolation (social alienation OR social isolation OR social distance OR isolation OR loneliness OR social connectivity OR social environment), older adults (aged OR older adult* OR older person*OR aged, 80 and over OR geriatric OR older senior) with an intervention focus (intervention* OR program* OR education* OR treatment* OR behavio* therapy OR health promotion*). Search criteria targeted peer-reviewed articles in English, restricted to older adults and to randomised controlled trials. The complete search strategy for one database is shown in Additional file 1, Supplementary Table 1. In addition, reference lists of eligible papers were screened for relevant articles.

### Study selection

**Fig. 1:**
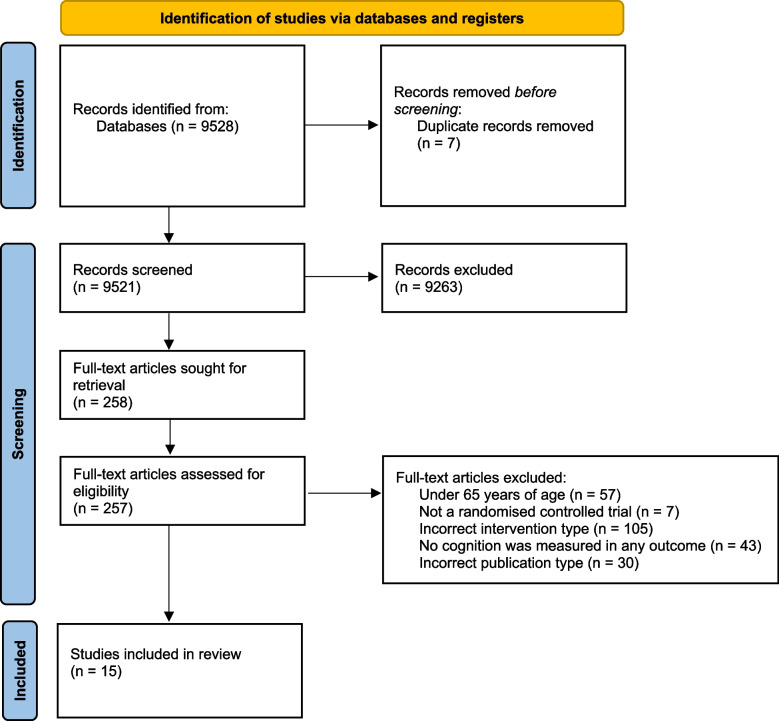
PRISMA diagram

Potential studies were exported into Rayyan with duplicates deleted by the primary author (JS). The initial screening of article titles and abstracts was conducted for the first 500 articles by three researchers (JS, VC, MA) to verify eligibility and calculate inter-rater reliability (95%). All discrepancies were discussed with the larger research team and resolved by consensus. The remainder of the papers were screened at title and abstract level by two researchers (VC, MA) independently. Inclusion of full text articles was completed independently by two research members (VC, MA) and checked by the primary author (JS). All steps of paper identification and selection are presented in the Preferred Reporting Items for Systematic Reviews and Meta- Analysis (PRISMA) diagram (Fig. 1).

### Data extraction

Data from eligible studies were extracted independently by three reviewers (VC, MA, MJRP) and verified by the primary author (JS). We used a pre-designed data extraction sheet to allow standardised reporting of results across studies, including information on: (1) study characteristics (e.g., study design, year, country, population group), (2) participant characteristics, (3) assessments used, (4) outcomes, and (5) study findings.

### BCTs coding and assumptions

Coding of the behaviour change techniques identified in the eligible studies was completed using established BCT taxonomies [46] and strategies described in previous research [53]. BCTs were coded where there was clear evidence of their application in the interventions described. The coders (VC and MJRP) received training from the research team members who are experienced in using BCT taxonomies and in the development of interventions for health promotion (FM) and implementation science (JS). Coders rated BCTs as either present or absent in the intervention arms separately with two rounds of separate coding required to achieve a suitable inter-rater agreement (first round, 84%, second round 95%). Discrepancies were brought to the research team, resolved through discussion until consensus was met, and updated accordingly. During the coding process, all interventions were scored for the presence of BCTs using the definition that BCTs aimed to modify participants' behaviour. For instance, a program aimed at enhancing social interaction among older adults may include established BCTs such as providing social support, setting social goals, and facilitating group discussions. These would be scored as present within this intervention because they are intended to influence individuals' behaviours towards engaging in more social interactions, the target of the intervention. Furthermore, in instances where interventions were described as provision of education or information without further detail, it was presumed that any informational session within an implementation intervention would encompass, at the very least, two BCTs: *offering information on consequences* and *instruction on behaviour execution*. However, additional BCTs were identified if observed. Finally, when interventions were described as providing "training" without further detail, it was inferred that, unless explicitly stated otherwise, any intervention featuring a training session would incorporate *instruction on behaviour execution*. A list of BCTs and their definition, as well as resulting codes from each study is provided in Additional file 1, Supplementary Table 2.

### Risk of bias

Study rigour was measured through risk of bias ratings of included studies which was conducted by three members (JS, VC, MJRP) of the research team using the Cochrane tool for assessing Risk of Bias in randomised trials (RoB2 tool, [54]) and confirmed by the primary author (JS) for further validation. The risk of bias tool covers the following domains of bias: (a) selection bias which includes sequence generation and allocation concealment, (b) performance bias which detects the blinding of participants and personnel, (c) blinding of outcome assessment, (d) attrition bias, and (e) reporting bias to determine level of bias (using a traffic light system of high, low or some concern). At the end, the overall risk of bias was set as low if the study was judged to be at low risk of bias for all domains [54]. A study was rated as some concerns if the study was judged to raise some concerns in at least one domain for this result, but not to be at high risk of bias for any domain. Studies with high risk of bias were those judged to be at high risk of bias in at least one domain, or the study was judged to have some concerns for multiple domains in a way that substantially lowered confidence in the results.

### Data analysis

Studies were grouped by intervention type and cognitive outcomes. Outcome measures that were assessed in at least two eligible RCTs using the same intervention (*cf.* control condition) were included in a separate meta-analysis [55, 56]. Reported outcome measures that were continuous in nature were translated to a standardised effect size (Hedges’ *g* = (m_i_-m_c_)/sd_ic_). RevMan (v5.3) was used to produce d and SE_d_, and forest plots, and estimates of the pooled effect and heterogeneity index *I*^2^ across the four outcomes (whereby 50–90% was considered as representing substantial heterogeneity). RevMan uses study sample size to weight effect sizes in a meta-analysis. Given the use of various outcome measures and intervention designs, it was improbable for our included studies to have shared an identical true effect size and thus a random-effects model was adopted. We reported on data closest to the intervention’s endpoint. Statistical significance of meta-analysis was set at *p*<0.05.

To investigate heterogeneity in main effects analyses, moderator effects of BCTs were explored using restricted maximum likelihood random effects meta-regressions. Univariate meta-regressions were carried out to examine the association between individual BCTs that were present (vs. not) and the effect of the number of BCTs used. Meta-regressions were only performed when there was evidence of substantial heterogeneity (*I*^*2*^ ≥ 50%), ≥ 10 trials per analysis [57], and at least four trials using a BCT, to minimise the impact of single trials.

## Results

### Overview

Database searches returned 9528 eligible studies for title and abstract screening, with fifteen articles ultimately deemed eligible from full-text screening and included in our final synthesis which is described from hereon [58–72] (Fig. 1).

Fifteen trials with 1,785 participants (range 20–348) were included in this review. Participants across the trials had a mean age of 76 years (range= 68.0–83.5 years), and the majority were women (70.6%, *n*=1261). Studies were conducted in the United States (*n*=2), Japan (*n*=4), China (*n*=1), Finland (*n*=1), Denmark (*n*=1), Ireland (*n*=1), Australia (*n*=1), Brazil (*n*=1), Spain (*n*=2) and Germany (*n*=1) (Table 1). The interventions took place mostly in the community (*n*=9, 60%) [59–72]. Table 1 further describes summary characteristics of included trial populations and detailed information about each trial included in this analysis, including all reported outcomes.

**Table 1:** Summary of included studies (*n*=15)

**Author /Year** **Country**	**Participant mean age** **(SD), Gender (% female)**	**Sample Size**	**Total retention rate**	**Time points data collection**	**Outcome(s)**	**Outcome(s) measure**
Ahessy 2016 [58] Ireland	83.5 years (SD=4.9) 86% female	Int N 20 Con N 20	90%	Weekly for 12 weeks	Global cognition Quality of life	MMSE CSDD CBS
Akanuma 2010 [59] Japan	78.0 years (SD=4.9) 77–88% female	Int N 12 Con N 12	-	0, 3 months	Global function Depression Behavioural condition Metabolic function	MMSE GDS BRSE CT / MRI
Dodge 2015 [60] USA	80.5 years (SD=6.8) 75.9% female	Int 1 N 24 Con 1 N 25 Int 2 N 17 Con 2 N 17	100%	0, 2, 12 weeks	Executive function Learning and memory Depression	Letter fluency (F, A, S), TMT‐A, TMT‐B, Category fluency (Animals), Stroop test Word list acquisition, Word list delayed recall, One, two back accuracy GDS-15
Gudex 2010 [61] Denmark	82.3 years (SD=9.7) 68% female	Int N 171 Con N 177	68%	0, 6, 12 months	Global cognition General functioning Agitated Behavior Quality of life	MMSE, SIB-S GBS CMAI ADRQoL
Iizuka 2018 [62] Japan	76.7 years (SD=4.5) 75% female	Int 1 N 27 Int 2 N 26 Con N 28	88.8%	0, 12 weeks	Global cognition Wellbeing Visual working memory Verbal working memory Immediate/delayed memory recall Executive function	MMSE‐J, MoCA‐J WHO‐5‐J VMST DST LM I / LM II TMT‐A, TMT‐B, CF
Meléndez-Moral 2013 [63] Spain	79.8 years (SD=9.3) 83.3% female	Int N 17 Con N 17	-	0, 3 months	General cognition Depression Self-esteem Life satisfaction Well-being	MMSE GDS RSS/RSES PGCMS RS
Mortimer 2012 [64] China	69.5 years (SD=5.8) 66.6% female	Int 1 N 30 Int 2 N 30 Int 3 N 30 Con 1 N 30	89 %	0, 20, 40 weeks	General cognition Executive function Confrontation naming Visual selective focused attention Visual memory Immediate & delayed memory Whole brain volume Number of steps	DRS CDT, Stroop Test (color-word), TMT‐A, TMT‐B, CFT, WAIS-R BNT Bell cancellation Test ROCF AVLT MRI Pedometer
Nakatsuka 2015 [65] Japan	81.5 years (SD=3.9) 54% female	Int 1 N 45 Int 2 N 38 Int 3 N 44	74.8 %	0, 12 weeks	Global cognition Executive function Depression Wellbeing Physical ability Enjoyableness of the intervention	MMSE, CDR WF, TMT-A GDS QOL 6-meter walk time PRO
Park 2014 [66] USA	71.6 years (SD=7.3) 73.9% female	Int 1 N 29 Int 2 N 35 Int 3 N 42 Con 1 N 36 Con 2 N 39 Con 3 N 40	85%	0, 12 weeks	Processing speed Mental control Episodic memory Visuospatial processing	Digit-comparison tasks with three, six, and nine items Cogstate Identification Flanker Center Letter, Arrow and Symbol tasks CANTAB (VRM) / HVLT (immediate, delayed) CANTAB (SWM), SPM
Peña 2014 [67] Spain	68.0 years (SD=6.4) 39% female	Int N 22 Con N 22	95%	0, 3 months	Patients’ premorbid IQ Global cognition Parkinson’s disease Depression Neuropsychiatric symptoms Functional disability Apathy	TAP MMSE UPDRS GDS NPI-Q WHO-DAS II LARS
Pitkala 2011 [68] Finland	80.0 years (SD=3.6) 73.6% female	Int 1 N 24 Con 1 N 24 Int 2 N 46 Con 2 N 46 Int 3 N 47 Con 3 N 48	88%	0, 3, 6, 12 months	Global cognition Depression	ADAS–Cog 15D
Tanaka 2012 [69] Japan	73.4 years (SD=4.9) 100% female	Int N 20 Con N 20	85%	0, 4, 8 weeks	Global cognition Depression	MMSE GDS-15
Tesky 2011 [70] Germany	72.0 years (SD=7.0) 73% female	Int 1 N 74 Int 2 N 56 Con N 78	78%	1, 12, 32 weeks	Global cognition Executive function	MMSE / ADAS–Cog / CDR / SDS TMT
Vidovich 2015 [71] Australia	75.0 (SD=5.8) 43% female	Int N 80 Con N 80	80%	0, 10, 52, 104 weeks	Global cognition Memory Attention Executive function	MMSE CAMCOG-R CVLT-II WAIS-R, COWAT
Zimmermann 2014 [72] Brazil	68.2 years (SD=3.8) **-**	Int N 10 Con N 10	70%	Weekly for 12 weeks	Depression Global cognition Attention Communication Executive function	GDS-15/ NEUPSILIN MMSE WAIS-III MCEB FDG–PET scans

*Abbreviations: 15D* 15 Dimensions Measures of Health Related Quality of Life, *ADAS–Cog* Alzheimer’s Disease Assessment Scale, *ADRQL* Alzheimer Disease Related Quality of Life, *AVLT* Auditory Verbal Learning Test, *BNT* Boston Naming Test, *CAMCI* Computer Assessment of Mild Cognitive Impairment, *CANTAB* Cambridge Neuropsychological Test Automated Battery, *CCI* Charlson Comorbidity Index, *CDR* Clinical Dementia Rating, *CDT* Clock-Drawing Test, *CFT* Category Verbal Fluency, *CMAI* Cohen-Mansfield Agitation Inventory, *Con* Control Group, *DRS* Mattis Dementia Rating Scale, *DST* The Digit Span Test, *GBS* Gottfries-Bråne-Steen scale, *GDS-15* Geriatric Depression Scale, *Int* Intervention Group, *LM* The Logical Memory I (immediate) II (delayed), *MMSE* Mini Mental State Examination, *MoCA* The Montreal Cognitive Assessment, *MRI* Magnetic Resonance Imaging, *NEO Big-5* Personality Inventory, *O* Objective Measure, *PRO* Patient-Reported Outcome, *QOL* Quality of Life, *RCT* Randomized Controlled Trial, *ROCF* Rey-Osterrieth Complex Figure, *S* Self-reported Data, *SD* Standard Deviation, *SIB-S* Severe Impairment Battery – Short Form, *SPM* Raven’s Standard Progressive Matrices, *SWM* Spatial Working Memory, *TMT-A* Trail Making Test A, *TMT-B* Trail Making Test B, *VMST* Visual Memory Span Test, *VRM* Verbal Recognition Memory Task, *WAIS-R* Wechsler Adult Intelligence Scale, *WF* Word Fluency, *WHO‐5‐J* Five Well‐Being Index Japanese version

### Interventions

Table 2 describes the summary characteristics of each intervention group. Social-based behavioural programs all aimed at building social connections, cognitive stimulation, and emotional well-being in older adults. The most common program types included reminiscence activities (e.g., group reminiscence approach, reality orientation; *n*=4, 26.7%) [59, 61, 63, 65], arts-based group endeavours (e.g., group choir music, quilt making, activities and discussions around art and therapeutic writing, photography; *n*=4, 26.7%) [58, 68, 72] and social-based communication (e.g. video chats, assignment of a communication robot; *n*=4, 26.7%) [60, 64, 67, 69], followed by cognitive-based group tasks (e.g. board games and puzzles; *n*=2, 13.3%) [62, 65] and education or social awareness building programs (*n*=2, 13.3%) [70, 71]. All except for one study [60] delivered group interventions face-to-face.

**Table 2:** Intervention characteristics

**First author** **year**	**Intervention type**	**Aim**	**Activity**	**Source of delivery** **Treatment setting**	**Duration (total dose)**	**Control group**	**Format**
Ahessy 2016 [58]	Arts-based	To explore whether participation in a music therapy choir intervention could reduce depression, quality of life and cognitive function in older adults	Choir session facilitated by a music therapist covering meditation and relaxation,vocal improvisation, singing and articulation exercises, and learning and singing repertoire/	Music therapist, researcher. Long-term residential units and day-care centre in Dublin.	1h weekly x 12 weeks (12 hours)	Standard nursing care	Face to face Group
Akanuma 2011 [59]	Reminiscence	To investigate the effect of psychosocial intervention on daily lives for vascular dementia	Group reminiscence focused on talking about topics related to the participants’ past such as childhood memories (toys, school days, textbooks), and epoch making events in one’s life (marriage, jobs)	Registered nurses, psychologists, speech therapists, occupational therapists. Geriatric nursing home in Nakada, Japan.	1h weekly x 12 weeks (12 hours)	Only supportive care	Face to face Group
Dodge 2015 [60]	Social-based	To assess adherence rates and effect of conversation-based cognitive stimulation through personal computers, webcams and a user-friendly interactive Internet interface on cognitive function	Face to face conversations with trained interviewers via video call	Trained research associates, interviewers trained research nurses, technical support personnel, single professional transcriber. Retirement communities and senior centers located in Portland, USA.	5 x 30–35 minute weekly x 6 weeks (15 hours)	Weekly telephone interview	Internet based conversation (Videochat)
Gudex 2010 [61]	Reminiscence	To strengthen individual’s identify, self-work, coherence and control of one’s own life	Reminiscence forms: general (group session for two to eight residents with similar backgrounds or interests), specific (tailored sessions for one to two residents focusing on individual communication needs) and spontaneous (informal use of comments during daily activities to elicit residents’ memories)	A reminiscence trainer, nursing staff. Danish nursing homes.	48 weeks	Usual care	Face to face Individual and Group
Iizuka 2018 [62]	Cognitive group-based	To clarify the influence of social interaction on the effect of a cognitive intervention program using Go	Activity involved attending a lecture on basic Go rules and techniques, solving Go exercises, learning tactics using a model game (kifu-narabe) and playing Go with other participants. The participants were also allowed to interact with instructors and other participants during the lessons and games and share feedback at the end	Four instructors. Community center in Tokyo, Japan.	1h weekly game x 12 weeks and 1h homework x 6 days (18 hours)	Health education	Face to face Group Tablet Individually
Meléndez-Moral 2013 [63]	Reminiscence	To investigate the usefulness of a reminiscence intervention in institutionalised care	Sessions focused on reminiscence themes such as childhood memories, hometown, games, songs, holidays, movies and family	Directed by a psychologist. Two retirement homes in the province of Valencia.	8h	Normal participation of activities found in nursing homes	Face to face Group
Mortimer 2012 [64]	Social-based	To assess the effect of social interventions on the risk of dementia, cognitive decline, or changes in brain volume	The social interaction group convened with a group leader and assistant, initially provided with guidance on discussion topics. However, participants independently opted to organise and select their own subjects	Medical personnel, group leaders and assistance, study coordinator. Jingansi Temple Community of Shanghai based on a government-maintained census name list.	3 x 50mins weekly x 40 weeks (~120 hours)	Phone contact four times	Face to face Group
Nakatsuka 2015 [65]	Reminiscence and cognitive group-based	To directly compare the effects of cognitive interventions, physical activities and group reminiscence approach	Cognitive group engaged in cognitive tasks and games targeting executive function and attention. The hysical activity group performed exercises such as walking and step aerobics and the reminiscence group participated in reality orientation and reminiscence discussions about past events and experiences. All three group sessions included a tea break and instructions for home assignments which were to be completed with family members	Medical doctors and public health nurses, certified neurologists, two raters, one instructor and two assistants. Public halls or community centers of the regions of living of the participants in Kurihara City, northern Japan.	1h weekly x 12 weeks (12 hours)	—	Face to face Group
Park 2014 [66]	Arts-based	To test whether sustained engagement in learning new skills activated working memory, episodic memory, and reasoning over a period of 3 months would enhance cognitive function in older adults	Structured training on respective skills on photography, computer, quilt making, digital photography and social group	Professional photographer, professional quilting instructor, instructor directed activities. Synapse Center located in a strip mall in Dallas, Texas.	15h weekly x 12 weeks (180 hours)	2) placebo condition 3) no-treatment condition.	Face to face Group
Peña 2014 [67]	Social-based	To examine the efficacy of an integrative cognitive training program (REHACOP) to improve cognition, clinical symptoms, and functional disability of patients with Parkinson disease	Structured paper-pencil task-based program that focused on restoration, compensation and optimisation strategies of rehabilitation	Neurologist and ASPARBI, psychologists. Department of Neurology at Galdakao Hospital and the Parkinson’s Disease Association (ASPARBI) both in Biscay.	3h weekly x 9 weeks (27 hours)	Occupational group activities conducted by a psychologist	Face to face Group
Pitkala 2011 [68]	Arts-based	To determine the effects of socially stimulating group intervention on cognition among older individuals reporting loneliness	Included socially stimulating activities and art experiences, group exercise and therapeutic writing to enhance communication, peer support and empowerment among participants	Registered nurses, occupational therapists, physiotherapists. Six communities from the Finnish National Population Register in seven study sites throughout Finland.	6h weekly x 12 weeks (72 hours)	Normal community care	Face to face Group
Tanaka 2012 [69]	Social-based	To investigate the effects of living with a communication robot on cognitive function and various physiological parameters in older women living alone	The communication robot which resembled a three-year-old boy, was programmed to engage in communication with the participants while the control robot had similar physical features but no verbal interaction.	Communication or control robot distributed by Kabochan Nodding Communication ROBOT. 40 older women living alone in their own homes in Osaka, Japan.	24h x 8 weeks (1344 hours)	Control robot was not designed to talk or nod.	Face to face Group
Tesky 2011 [70]	Education/ awareness building	To investigate the effects of leisure activities on cognitive performance of healthy older subjects	The intervention groups received training in the AKTIVA program, but the second intervention group received additional nutritional education and a physical exercise program. Participants in the second intervention group also underwent physical check-ups, introductory courses in various physical activities, a nutrition workshop and were required to maintain a movement diary.	— Senior social clubs and community centers,	1x weekly + 2 booster sessions x 8 weeks	Booklet pertaining to the training topics at the end of intervention	Face to face Group
Vidovich 2015 [71]	Education/ awareness building	To clarify whether a group cognitive activity strategy training program would decrease the 2-year rate of cognitive decline of people with mild cognitive impairment	Program focused on age-associated changes in cognition and provided activities to enhance attention, memory, and executive function. The sessions incorporated cognitive rehabilitation, stimulation, and training.	Clinical neuropsychologist and research assistants Community volunteers living with mild cognitive impairment.	2x weekly (15 hours)	A 5-week program or more generalized presentations on healthy ageing and retirement	Face to face Group
Zimmermann 2014 [72]	Arts-based	To verify whether differences between two approaches (structured working memory program, poetry-based simulation program) exists.	Working memory sessions emphasised on basic processing components initially but progressively increased to incorporate more demanding abilities over time. With poetry-based stimulation, which was led by a professor and a student of languages and literature, focused on improving reading and interpretation abilities of poetry. The participants read poems, listened to songs, visualised related pictures and discussed subjective meanings and main ideas.	Clinicians, psychologist students, professor, student of Languages and Literature. —	1 x weekly (12 hours)	—	Face to face Group

On average, the interventions lasted 28 weeks (range 6–48 weeks), with an average retention rate of 84% (range 68–100). Apart from three studies [61, 63, 69], all studies had interventions that were delivered at least once every week.

### Outcomes

The majority of the studies measured global cognitive function using self-reported tools (*n* = 14; with the clinician-administered Mini-Mental Status Examination most commonly reported (MMSE, *n* = 11)). Several of the studies also assessed executive function (*n* = 7), which often used the Trail Making Test (TMT-A (*n* = 3); TMT-B (*n*=2)). Two studies measured quality of life using self-reported measures (e.g., Quality of life Face Scale Score).

**Fig. 2:**
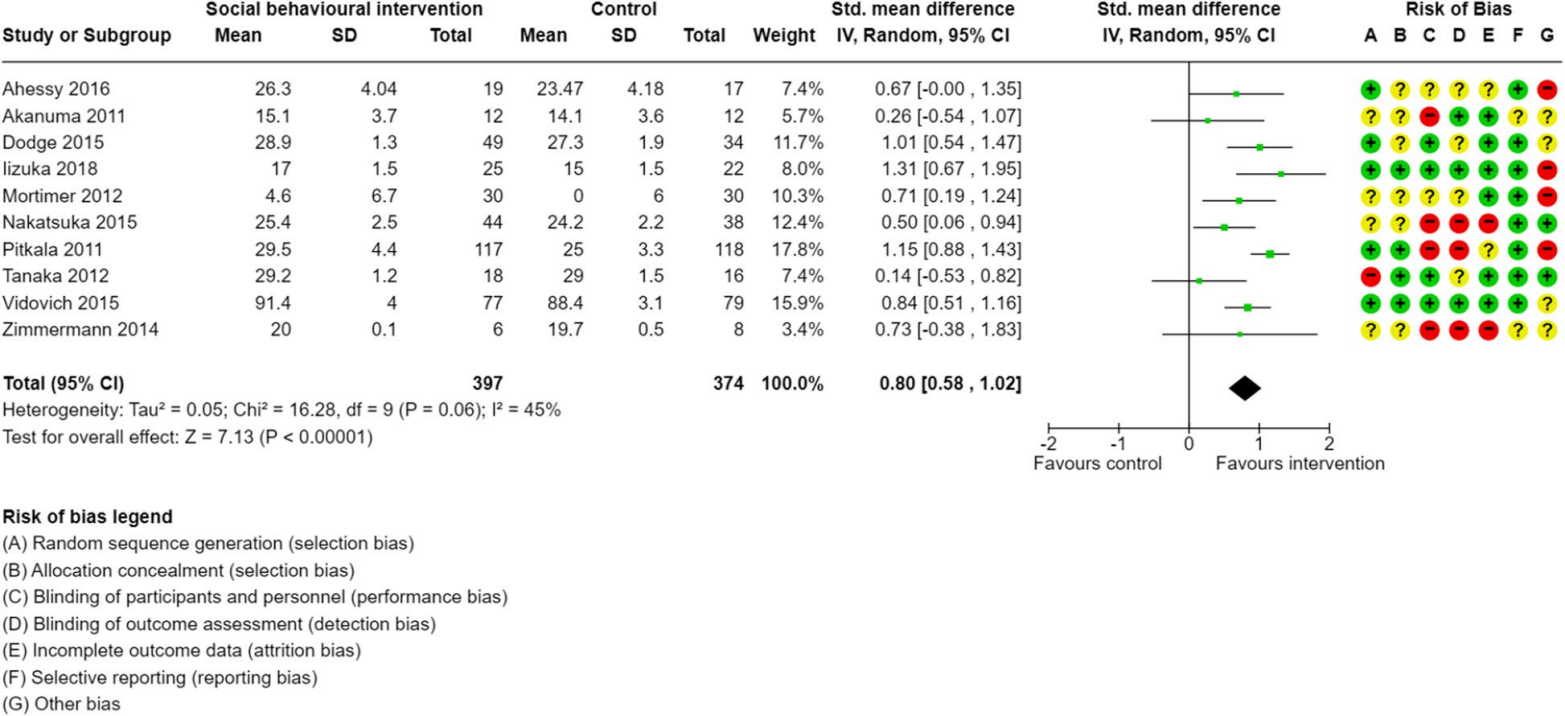
Forest plot of social interventions and effects on global cognition

**Fig. 3:**
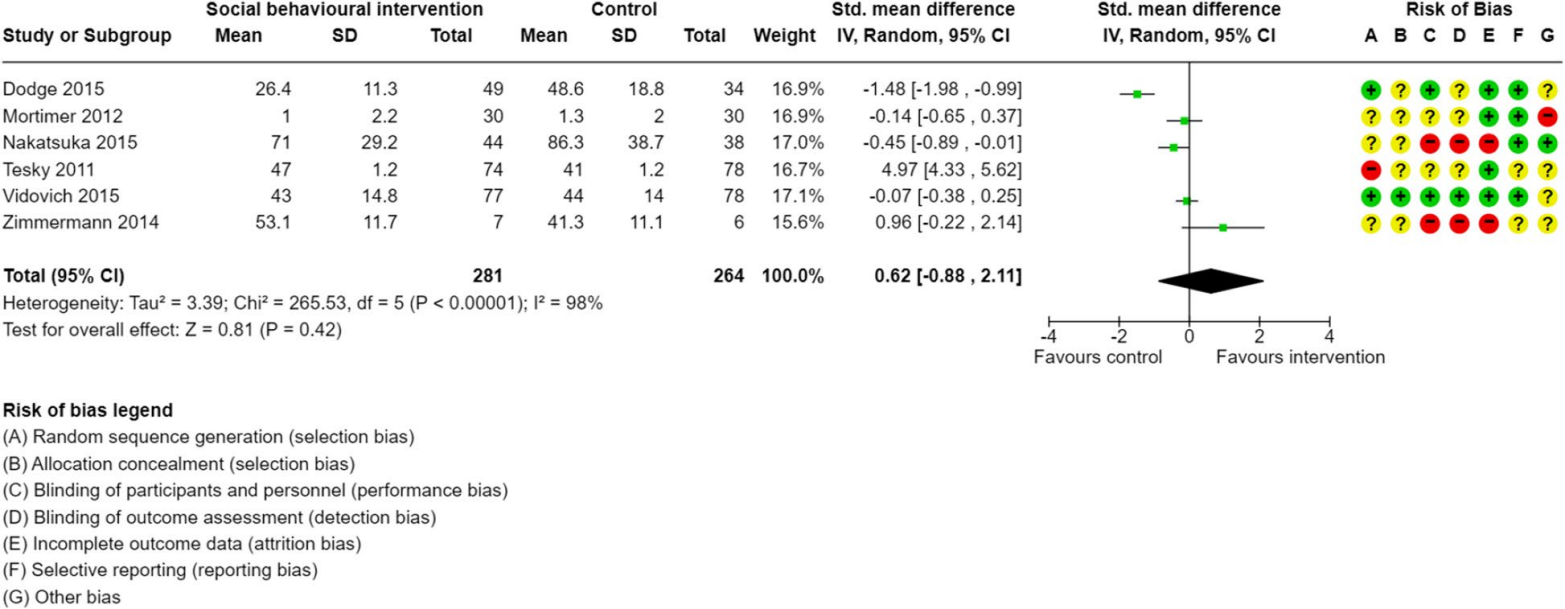
Forest plot of social interventions and effects on executive function

Figures 2 and 3 present the results of the random-effects meta-analysis estimating the mean change from pre-intervention to post-intervention for global cognition and executive function. Of the studies included, 10 provided sufficient data for inclusion in the meta-analysis for global cognition, and 6 for executive function. Overall, social-based behavioural interventions significantly improved global cognition (*d* = 0.80, 95% CI: [0.58, 1.02], k = 10, *p* < 0.001) with moderate heterogeneity (*I*^2^=45%) (Fig. 2). Social-based behavioural interventions had no significant effects on executive function (*d* = 0.62, 95% CI: [−0.88, 2.11], k = 6, *p* = 0.42) (Fig. 3).

In the three studies that scored acceptable in five or more risk of bias dimensions [62, 69, 71], two reported beneficial changes in global cognition [62, 71].

### Behaviour change techniques

Table 3 shows the BCTs coded for each of the 15 studies. Out of 93 possible BCTs, we identified 20 BCTs (21.5%) targeting changes in the social behaviour of participants. Out of 16 possible BCT categories, 12 categories (75%) were coded, with ‘Associations’, ‘Reward and threat’, ‘Scheduled consequences’, and ‘Covert learning’ not included. The most commonly applied BCTs targeting behaviours included: 1) goal setting (15/15, 100%); 2) action planning (15/15, 100%); 3) instruction on how to perform a behaviour (15/15, 100%); 4) demonstration of the behaviour (8/15, 53.3%); 5) monitoring of emotional consequences (6/15, 40%); 6) credible source (6/15, 40%), and 7) adding objects to the environment (6/15, 40%). The average number of BCTs used in each study were 6 BCTs (range 3–15).

**Table 3:**
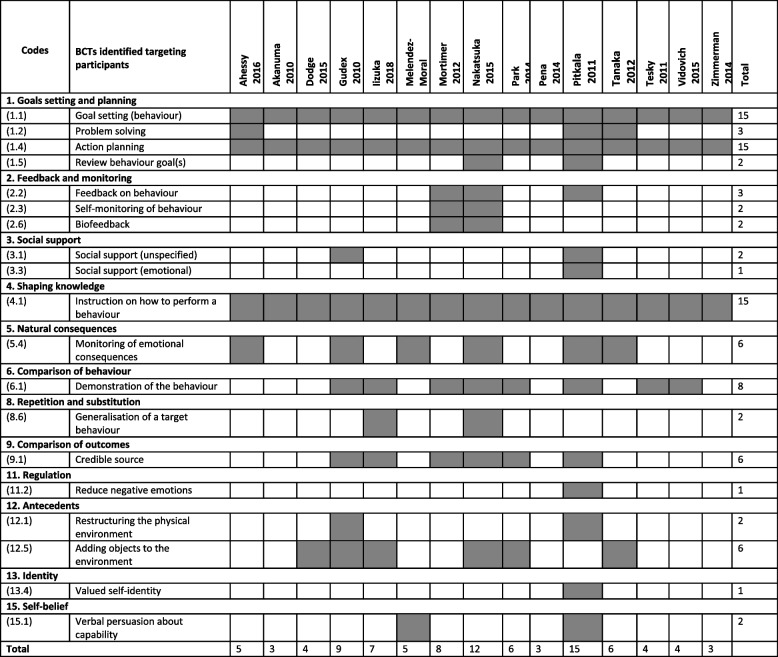
Behaviour change techniques identified in the included studies

A number of BCTs were identified in the five studies that showed benefits on global cognition. Major differences found between identified BCTs in effective compared to non-effective interventions indicated that most popular BCTs of feedback and self-monitoring of behaviour were associated with positive intervention effect (Table 3). Goal setting of behaviour, action planning, demonstration of behaviour and having credible sources were also identified, however these were also present in non-effective interventions. Demonstration of outcome (BCT6) was significantly associated with outcome effect (b = −0.602; 95% CI: −1.195 to −0.009). This model predicted 94.6% of the variance (Table 4).

**Table 4:** Results from meta-regression analysis of social-based behavioural interventions

**Study characteristics**	*ß*	**t**	**95% CI**	***p*-value**
Intercept	0.607	4.042	0.252 – 0.962	0.005
BCT6: Comparison of behaviour	−0.418	−2.869	−0.762 – −0.073	0.024
BCT12: Antecedents	0.150	1.115	−0.169 – 0.469	0.302
**Adjusted R**^**2**^** %**	**94.6**			

*Abbreviations and symbols: BCT* Behaviour change technique, *ß* Estimated meta-regression coefficient, *CI* Confidence interval, *Adj. R*^*2*^ Adjusted proportion of between study variance explained by predictors

### Methodological quality

**Fig. 4:**
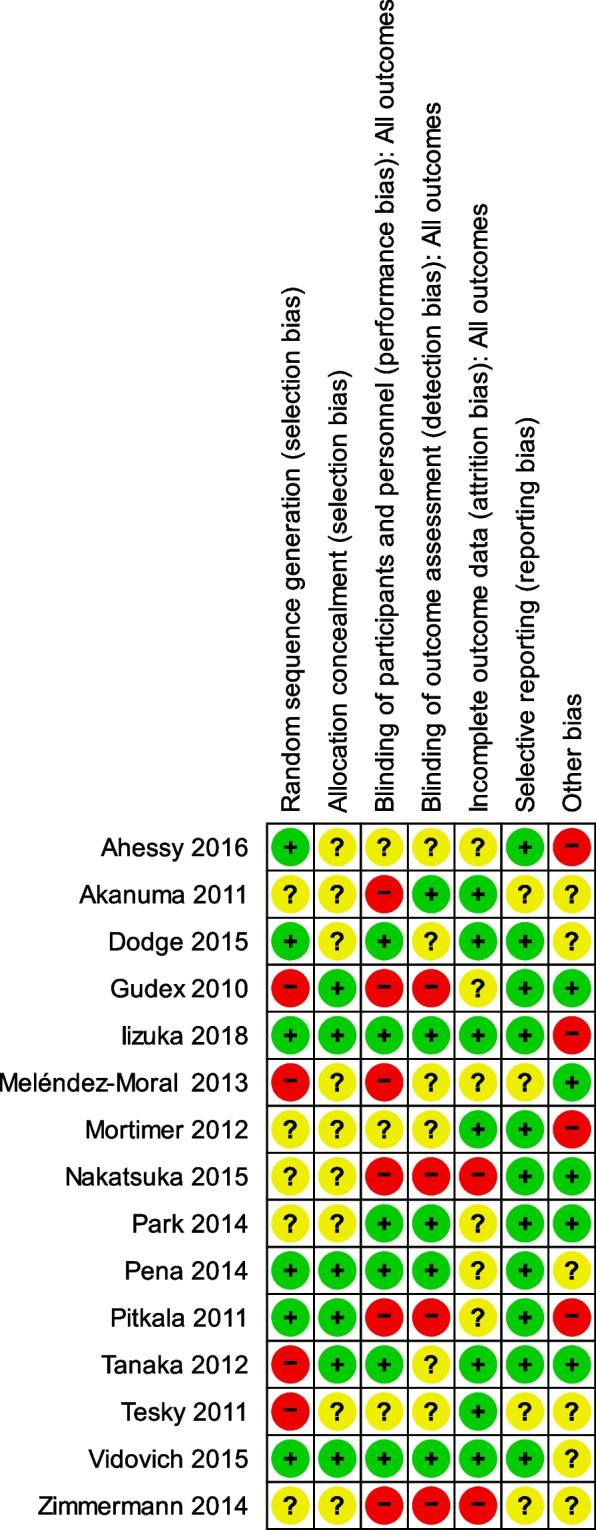
Risk of bias summary

The overall risk of bias was high for 14/15 studies (93.3%) (Fig. 4). The risk of bias was judged low for selective reporting in 11 of the studies (73.3%). Four studies (26.7%) were judged to be at a high risk of bias related to the lack of random sequence generation, 6 (40%) were judged to be at a high risk due to lack of concealed allocation and blinding of participants and personnel, 4 (26.7%) were judged to be at a high risk of bias related to lack of blinding of outcome assessment, 2 (13.3%) were judged to be at a high risk due to having incomplete outcome data, and 4 (26.7%) were assessed as susceptible to other potential sources of bias. Out of the 15 studies, only 1 (6.7%) had consistent low risk of bias in all six categories.

## Discussion

This systematic review and meta-analysis provided a comprehensive examination of behaviour change techniques applied in social interventions resulting in improved cognition in older adults. Three BCTs were identified to be present in all 15 studies (goal setting of the behaviour, action planning and instruction on how to perform a behaviour), however only the BCT of comparison of behaviour was associated with improved cognitive outcomes. Our analysis highlights the potential of interventions that increase social engagement to improve cognitive health among older adults, and emphasises a role for behaviour change techniques in achieving positive cognitive outcomes.

### Effectiveness of interventions: global cognition

Our findings suggest that social intervention programs can positively impact on global cognition, but not specifically on executive function. Multiple studies that investigated the impact of social activity on global cognition suggest that social intervention programs may be useful for promoting brain health in older adults. This is likely due to a boost in neuroplasticity affecting large-scale brain network connectivity and function, conferring generalised global cognitive benefits [73]. Larger social networks and greater levels of social support have also been associated with positive effects on global cognition [74]. However, there is a dual effect where higher levels of engagement promote positive cognitive outcome and better cognitive functioning is related to living a more engaged lifestyle [75, 76]. Having said this, in order to conduct a meta-analysis as we have done here, it was necessary to group interventions by common outcomes, rather than by the characteristics of the interventions themselves, particularly given the small number of studies identified that addressed our research question. Future work could seek to delineate the unique contributions of social interventions on a range of cognitive domains.

Social interactions are hypothesized to increase cognitive reserve through two pathways: bridging (provide cognitive stimulation via doing activities with others) and bonding (reduced stress via close relationships) [70]. It may be that certain types of social interactions have an influence on specific cognitive domains. The lack of association between social interventions and changes in specific cognitive domains may be related to differences in types of studies and the outcomes they included. For instance, only five studies assessed executive function, with most comprising of informal conversation groups. The only study to show a change in executive function was an arts based program which may have promoted planning skills [72]. Future research is needed to identify whether specific cognitive domains are influenced by specific kinds of social interventions.

Whilst social activities show an improvement in global cognition and increased brain volume, studies have highlighted that social intervention programs do not seem to affect specific cognitive domains such as memory, attention, or executive function [74]. A potential reason behind the contrasting outcomes observed between the influences of social support and engagement in social activities or networks could stem from the distinct role that social support plays in managing stress. Research indicates that social support contributes to building resilience against the detrimental effects of stress, which may have a buffering effect, helping to preserve cognition in older age [74]. In contrast, merely participating in social activities or having a broader circle of family and friends might not encompass the necessary social and emotional backing that is instrumental in reaping stress-alleviating advantages or the advantages of cognitive stimulation provided by doing activities which promote the use of specific cognitive skills.

### BCTs and social behaviour

The BCT “demonstration of behaviour” is considered a key element within behaviour change interventions and is often observed in various systematic reviews spanning different fields [77–80]. This BCT involves the provision of real-life examples or models of the desired behaviour, which serves to illustrate the achievable outcomes and establish a reference point for individuals. Usually, demonstrating the behaviour in a tangible way offers a clear visual representation that can stimulate motivation and provide a sense of attainability. Previous systematic reviews have consistently highlighted the presence and impact of this BCT. In health interventions, such as exercise promotion or dietary changes [81–84], presenting role models or showcasing individuals who have successfully adopted the desired behaviour has been found effective in motivating others to follow suit. The human tendency to learn from and emulate others’ actions amplifies the potential of this technique. However, the effectiveness of behaviour demonstration hinges on factors such as relatability and authenticity of the models, as well as individual perceptions [85, 86]. Overly idealised representations might lead to feelings of inadequacy or disbelief and can negate the intended impact [87, 88]. Future social-based programs should consider how behaviour demonstration could be carried out, and provide tangible, relatable examples of desired actions to bridge the gap between intention and execution. By providing real-world instances of successful behaviour adoption, interventions can tap into social influence dynamics and inspire individuals to be more socially engaged.

The effectiveness of goal setting has been established in various domains, and its presence and impact have also been documented in multiple systematic reviews across multiple contexts [81, 82, 89, 90]. However, we were unable to identify its role in our current review. The efficacy of goal setting is likely contingent on factors like goal specificity, realism, and individual characteristics [91, 92] and these varied in the included studies. Research indicates that overly ambitious goals can lead to frustration and non-compliance, while overly simple goals might not challenge individuals adequately [93]. Moreover, goal setting might not be equally effective across all social conditions; its impact could vary depending on factors such as the complexity of the targeted behaviour and the individual’s level of commitment [94–96]. As such, its integrative role in future programs suggests its versatility and potential applicability in combating social issues such as isolation or engagement. As social interventions strive to bolster connectivity and improve mental well-being, goal setting could provide a structured approach for individuals to establish and pursue social participation objectives. Nevertheless, identifying BCTs can support the assessment of interventions that target several outcomes [97]. Whilst the quantity of BCTs is not necessarily associated with better outcomes, combinations of BCTs might increase its effectiveness [97].

### Implications

Our review highlights a need for future interventions to prioritise robust BCT components and to acknowledge the potential impact of social interactions for promoting cognitive health among older adults. While the current meta-analysis offers new insights, the average duration of interventions remains short in nature (mean 23 months) and necessitates larger, longer-term trials. These lengthier trials would permit a more comprehensive understanding of the sustained effects of social-based interventions and facilitate additional explorations of the variability in outcomes and effectiveness of BCTs over extended periods. Such trials also need to be more robust in design to ensure compliance with randomisation sequences, blinding and full, not selective, reporting. Furthermore, given the observed variability in outcomes across demographic (e.g., gender and language) compositions, there is an additional implication for tailoring interventions based on demographic and cultural characteristics. Finally, we note that many studies did not include specific pre and post measures relating to social outcomes (e.g. social network size), and future research could examine whether the extent to which there are improvements in cognition is associated with measurable improvements on social measures. Policy and practice changes may need to incorporate specific considerations in the design and implementation of social interventions in order to enhance the efficacy of interventions and contribute to more equitable mental health outcomes among older adults.

### Limitations

This review had a limited number of studies suitable for meta-analysis and synthesis. By only including studies published in English, there is a possibility we limited the number of available interventions for this review (e.g., latest study was in 2018). Furthermore, the inclusion of studies where cognition served as a secondary outcome rather than the primary focus is an additional limitation. In these instances, the interventions may not have been adequately powered to detect significant effects on cognition, as their design, sample size and power calculations may have primarily targeted other outcomes. This, in combination with multiple BCTs used and a variety of outcome measures, makes it difficult to allocate an effect size to a specific BCT. Further, because social interventions contain multiple modules with interactive components, it is difficult to attribute an improvement in a particular study outcome to one specific BCT. Additionally, it is important to recognise the limitations inherent in the use of specific instruments (e.g., TMT-A) as a proxy for executive function. Whilst we included several tests to measure a composite outcome for executive function, this was done to broaden the scope of assessment and increase the variability in the measures, thereby strengthening the overall analytical robustness. This decision aligns with the methodological imperative of enhancing the precision of effect size estimates and ensuring the reliability of the findings. As such, because of the emerging nature of this research field, which is very much in its infancy, the meta-analyses and meta-regressions were substantially underpowered, and we found that the majority of included studies had a risk of bias. This should also be taken into consideration with the fact that many studies contained small sample sizes and are likely at risk of type 1 error. Future work should seek to replicate these findings in larger trials and implementation studies.

## Conclusion

There are a limited number of social-behavioural trials investigating the effectiveness of BCTs for the improvement of cognitive and mental health. The evidence to date supports the use of social interventions for improving global cognition in older adults, with comparison of behaviour being associated with positive changes. There is insufficient evidence linking social interventions to changes in specific cognitive domains, perhaps related to the discrepancy across interventions in the outcome measures they included. Moving forward, the findings advocate for the incorporation of social strategies into future interventions, emphasising their potential to yield clinically meaningful benefits for global cognition. Given these results, future research to identify the longer-term effects and sustainability of these approaches is warranted, which can contribute to a more comprehensive understanding of the enduring impact of social-based interventions on cognitive outcomes in older adults.

## Supplementary Information

Supplementary Material 1.

## Data Availability

No datasets were generated or analysed during the current study.
