# Supplementary material for: A meta-analysis of behaviour change techniques in social interventions targeting improved cognitive function in older adults
Source: BMC Public Health. 2025 Mar 27;25:1158. doi: 10.1186/s12889-025-22229-x (PMC11948784; doi:10.1186/s12889-025-22229-x)
Supplement: Supplementary file 1 — Supplementary Material 1. [file 12889_2025_22229_MOESM1_ESM.docx]

**Additional files**

**Supplementary Table 1. Search strategy**

PUBMED


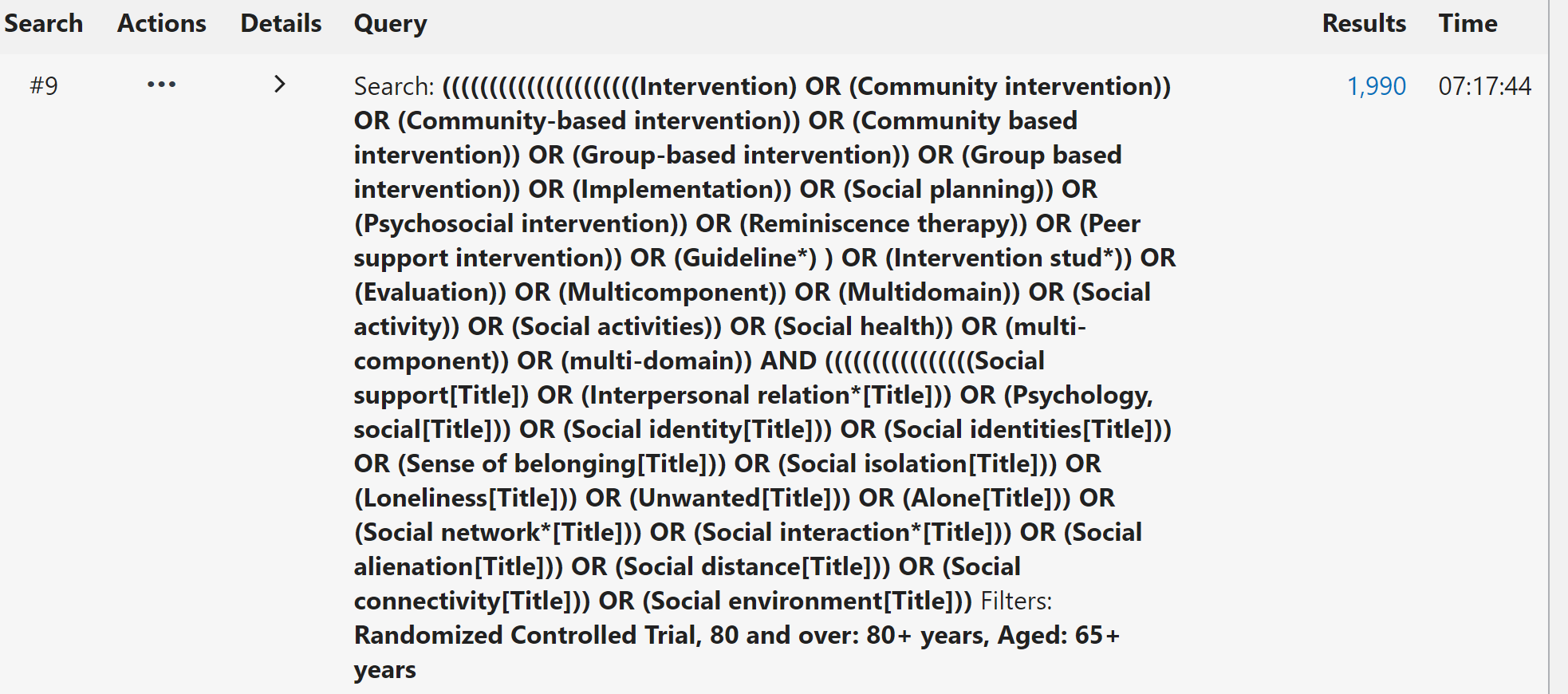


EMBASE

|  |  |  |  |  |  |  |
| --- | --- | --- | --- | --- | --- | --- |
| 37 |  from 35 keep 1001-2000 | 1000 | Advanced | [Display Results](https://ovidsp-dc2-ovid-com.simsrad.net.ocs.mq.edu.au/ovid-b/ovidweb.cgi?&S=OPBDFPHCDOEBHAEOIPNJJHLECAGIAA00&SELECT=S.sh%7c&R=37&Process+Action=display)  [More](https://ovidsp-dc2-ovid-com.simsrad.net.ocs.mq.edu.au/ovid-b/ovidweb.cgi?&S=OPBDFPHCDOEBHAEOIPNJJHLECAGIAA00&SELECT=S.sh%7c&R=35&Process+Action=display) |  |  |
|  | 36 |  from 28 keep 1001-2111 | 1111 | Advanced | [Display Results](https://ovidsp-dc2-ovid-com.simsrad.net.ocs.mq.edu.au/ovid-b/ovidweb.cgi?&S=OPBDFPHCDOEBHAEOIPNJJHLECAGIAA00&SELECT=S.sh%7c&R=36&Process+Action=display)  [More](https://ovidsp-dc2-ovid-com.simsrad.net.ocs.mq.edu.au/ovid-b/ovidweb.cgi?&S=OPBDFPHCDOEBHAEOIPNJJHLECAGIAA00&SELECT=S.sh%7c&R=35&Process+Action=display) |  |
|  | 35 |  limit 34 to (embase or medline) [Limit not valid in Journals@Ovid,Ovid MEDLINE(R),Ovid MEDLINE(R) Daily Update,Ovid MEDLINE(R) PubMed not MEDLINE,Ovid MEDLINE(R) In-Process,Ovid MEDLINE(R) Publisher,APA PsycInfo; records were retained] | 2111 | Advanced | [Display Results](https://ovidsp-dc2-ovid-com.simsrad.net.ocs.mq.edu.au/ovid-b/ovidweb.cgi?&S=OPBDFPHCDOEBHAEOIPNJJHLECAGIAA00&SELECT=S.sh%7c&R=35&Process+Action=display)  [More](https://ovidsp-dc2-ovid-com.simsrad.net.ocs.mq.edu.au/ovid-b/ovidweb.cgi?&S=OPBDFPHCDOEBHAEOIPNJJHLECAGIAA00&SELECT=S.sh%7c&R=35&Process+Action=display) |  |
|  | 34 |  limit 33 to human [Limit not valid in Journals@Ovid; records were retained] | 2111 | Advanced | [Display Results](https://ovidsp-dc2-ovid-com.simsrad.net.ocs.mq.edu.au/ovid-b/ovidweb.cgi?&S=OPBDFPHCDOEBHAEOIPNJJHLECAGIAA00&SELECT=S.sh%7c&R=34&Process+Action=display)  [More](https://ovidsp-dc2-ovid-com.simsrad.net.ocs.mq.edu.au/ovid-b/ovidweb.cgi?&S=OPBDFPHCDOEBHAEOIPNJJHLECAGIAA00&SELECT=S.sh%7c&R=35&Process+Action=display) |  |
|  | 33 |  limit 32 to (pubmed-not-medline or "pubmed/medline") [Limit not valid in Journals@Ovid,Ovid MEDLINE(R),Ovid MEDLINE(R) Daily Update,Ovid MEDLINE(R) PubMed not MEDLINE,Ovid MEDLINE(R) In-Process,Ovid MEDLINE(R) Publisher,APA PsycInfo; records were retained] | 2111 | Advanced | [Display Results](https://ovidsp-dc2-ovid-com.simsrad.net.ocs.mq.edu.au/ovid-b/ovidweb.cgi?&S=OPBDFPHCDOEBHAEOIPNJJHLECAGIAA00&SELECT=S.sh%7c&R=33&Process+Action=display)  [More](https://ovidsp-dc2-ovid-com.simsrad.net.ocs.mq.edu.au/ovid-b/ovidweb.cgi?&S=OPBDFPHCDOEBHAEOIPNJJHLECAGIAA00&SELECT=S.sh%7c&R=35&Process+Action=display) |  |
|  | 32 |  limit 31 to english | 2490 | Advanced | [Display Results](https://ovidsp-dc2-ovid-com.simsrad.net.ocs.mq.edu.au/ovid-b/ovidweb.cgi?&S=OPBDFPHCDOEBHAEOIPNJJHLECAGIAA00&SELECT=S.sh%7c&R=32&Process+Action=display)  [More](https://ovidsp-dc2-ovid-com.simsrad.net.ocs.mq.edu.au/ovid-b/ovidweb.cgi?&S=OPBDFPHCDOEBHAEOIPNJJHLECAGIAA00&SELECT=S.sh%7c&R=35&Process+Action=display) |  |
|  | 31 |  limit 30 to aged <65+ years> [Limit not valid in Journals@Ovid,Ovid MEDLINE(R),Ovid MEDLINE(R) Daily Update,Ovid MEDLINE(R) PubMed not MEDLINE,Ovid MEDLINE(R) In-Process,Ovid MEDLINE(R) Publisher; records were retained] | 2490 | Advanced | [Display Results](https://ovidsp-dc2-ovid-com.simsrad.net.ocs.mq.edu.au/ovid-b/ovidweb.cgi?&S=OPBDFPHCDOEBHAEOIPNJJHLECAGIAA00&SELECT=S.sh%7c&R=31&Process+Action=display)  [More](https://ovidsp-dc2-ovid-com.simsrad.net.ocs.mq.edu.au/ovid-b/ovidweb.cgi?&S=OPBDFPHCDOEBHAEOIPNJJHLECAGIAA00&SELECT=S.sh%7c&R=35&Process+Action=display) |  |
|  | 30 |  limit 29 to (clinical trial or randomized controlled trial or controlled clinical trial) [Limit not valid in Journals@Ovid,APA PsycInfo; records were retained] | 2815 | Advanced | [Display Results](https://ovidsp-dc2-ovid-com.simsrad.net.ocs.mq.edu.au/ovid-b/ovidweb.cgi?&S=OPBDFPHCDOEBHAEOIPNJJHLECAGIAA00&SELECT=S.sh%7c&R=30&Process+Action=display)  [More](https://ovidsp-dc2-ovid-com.simsrad.net.ocs.mq.edu.au/ovid-b/ovidweb.cgi?&S=OPBDFPHCDOEBHAEOIPNJJHLECAGIAA00&SELECT=S.sh%7c&R=35&Process+Action=display) |  |
|  | 29 |  limit 21 to "all aged (65 and over)" [Limit not valid in Journals@Ovid,Embase,APA PsycInfo; records were retained] | 5036 | Advanced | [Display Results](https://ovidsp-dc2-ovid-com.simsrad.net.ocs.mq.edu.au/ovid-b/ovidweb.cgi?&S=OPBDFPHCDOEBHAEOIPNJJHLECAGIAA00&SELECT=S.sh%7c&R=29&Process+Action=display)  [More](https://ovidsp-dc2-ovid-com.simsrad.net.ocs.mq.edu.au/ovid-b/ovidweb.cgi?&S=OPBDFPHCDOEBHAEOIPNJJHLECAGIAA00&SELECT=S.sh%7c&R=35&Process+Action=display) |  |
|  | 28 |  limit 27 to (embase or medline) [Limit not valid in Journals@Ovid,Ovid MEDLINE(R),Ovid MEDLINE(R) Daily Update,Ovid MEDLINE(R) PubMed not MEDLINE,Ovid MEDLINE(R) In-Process,Ovid MEDLINE(R) Publisher,APA PsycInfo; records were retained] | 2111 | Advanced | [Display Results](https://ovidsp-dc2-ovid-com.simsrad.net.ocs.mq.edu.au/ovid-b/ovidweb.cgi?&S=OPBDFPHCDOEBHAEOIPNJJHLECAGIAA00&SELECT=S.sh%7c&R=28&Process+Action=display)  [More](https://ovidsp-dc2-ovid-com.simsrad.net.ocs.mq.edu.au/ovid-b/ovidweb.cgi?&S=OPBDFPHCDOEBHAEOIPNJJHLECAGIAA00&SELECT=S.sh%7c&R=35&Process+Action=display) |  |
|  | 27 |  limit 26 to human [Limit not valid in Journals@Ovid; records were retained] | 2111 | Advanced | [Display Results](https://ovidsp-dc2-ovid-com.simsrad.net.ocs.mq.edu.au/ovid-b/ovidweb.cgi?&S=OPBDFPHCDOEBHAEOIPNJJHLECAGIAA00&SELECT=S.sh%7c&R=27&Process+Action=display)  [More](https://ovidsp-dc2-ovid-com.simsrad.net.ocs.mq.edu.au/ovid-b/ovidweb.cgi?&S=OPBDFPHCDOEBHAEOIPNJJHLECAGIAA00&SELECT=S.sh%7c&R=35&Process+Action=display) |  |
|  | 26 |  limit 25 to (pubmed-not-medline or "pubmed/medline") [Limit not valid in Journals@Ovid,Ovid MEDLINE(R),Ovid MEDLINE(R) Daily Update,Ovid MEDLINE(R) PubMed not MEDLINE,Ovid MEDLINE(R) In-Process,Ovid MEDLINE(R) Publisher,APA PsycInfo; records were retained] | 2111 | Advanced | [Display Results](https://ovidsp-dc2-ovid-com.simsrad.net.ocs.mq.edu.au/ovid-b/ovidweb.cgi?&S=OPBDFPHCDOEBHAEOIPNJJHLECAGIAA00&SELECT=S.sh%7c&R=26&Process+Action=display)  [More](https://ovidsp-dc2-ovid-com.simsrad.net.ocs.mq.edu.au/ovid-b/ovidweb.cgi?&S=OPBDFPHCDOEBHAEOIPNJJHLECAGIAA00&SELECT=S.sh%7c&R=35&Process+Action=display) |  |
|  | 25 |  limit 24 to english | 2490 | Advanced | [Display Results](https://ovidsp-dc2-ovid-com.simsrad.net.ocs.mq.edu.au/ovid-b/ovidweb.cgi?&S=OPBDFPHCDOEBHAEOIPNJJHLECAGIAA00&SELECT=S.sh%7c&R=25&Process+Action=display)  [More](https://ovidsp-dc2-ovid-com.simsrad.net.ocs.mq.edu.au/ovid-b/ovidweb.cgi?&S=OPBDFPHCDOEBHAEOIPNJJHLECAGIAA00&SELECT=S.sh%7c&R=35&Process+Action=display) |  |
|  | 24 |  limit 23 to aged <65+ years> [Limit not valid in Journals@Ovid,Ovid MEDLINE(R),Ovid MEDLINE(R) Daily Update,Ovid MEDLINE(R) PubMed not MEDLINE,Ovid MEDLINE(R) In-Process,Ovid MEDLINE(R) Publisher; records were retained] | 2490 | Advanced | [Display Results](https://ovidsp-dc2-ovid-com.simsrad.net.ocs.mq.edu.au/ovid-b/ovidweb.cgi?&S=OPBDFPHCDOEBHAEOIPNJJHLECAGIAA00&SELECT=S.sh%7c&R=24&Process+Action=display)  [More](https://ovidsp-dc2-ovid-com.simsrad.net.ocs.mq.edu.au/ovid-b/ovidweb.cgi?&S=OPBDFPHCDOEBHAEOIPNJJHLECAGIAA00&SELECT=S.sh%7c&R=35&Process+Action=display) |  |
|  | 23 |  limit 22 to (clinical trial or randomized controlled trial or controlled clinical trial) [Limit not valid in Journals@Ovid,APA PsycInfo; records were retained] | 2815 | Advanced | [Display Results](https://ovidsp-dc2-ovid-com.simsrad.net.ocs.mq.edu.au/ovid-b/ovidweb.cgi?&S=OPBDFPHCDOEBHAEOIPNJJHLECAGIAA00&SELECT=S.sh%7c&R=23&Process+Action=display)  [More](https://ovidsp-dc2-ovid-com.simsrad.net.ocs.mq.edu.au/ovid-b/ovidweb.cgi?&S=OPBDFPHCDOEBHAEOIPNJJHLECAGIAA00&SELECT=S.sh%7c&R=35&Process+Action=display) |  |
|  | 22 |  limit 21 to "all aged (65 and over)" [Limit not valid in Journals@Ovid,Embase,APA PsycInfo; records were retained] | 5036 | Advanced | [Display Results](https://ovidsp-dc2-ovid-com.simsrad.net.ocs.mq.edu.au/ovid-b/ovidweb.cgi?&S=OPBDFPHCDOEBHAEOIPNJJHLECAGIAA00&SELECT=S.sh%7c&R=22&Process+Action=display)  [More](https://ovidsp-dc2-ovid-com.simsrad.net.ocs.mq.edu.au/ovid-b/ovidweb.cgi?&S=OPBDFPHCDOEBHAEOIPNJJHLECAGIAA00&SELECT=S.sh%7c&R=35&Process+Action=display) |  |
|  | 21 |  limit 20 to human [Limit not valid in Journals@Ovid; records were retained] | 5115 | Advanced | [Display Results](https://ovidsp-dc2-ovid-com.simsrad.net.ocs.mq.edu.au/ovid-b/ovidweb.cgi?&S=OPBDFPHCDOEBHAEOIPNJJHLECAGIAA00&SELECT=S.sh%7c&R=21&Process+Action=display)  [More](https://ovidsp-dc2-ovid-com.simsrad.net.ocs.mq.edu.au/ovid-b/ovidweb.cgi?&S=OPBDFPHCDOEBHAEOIPNJJHLECAGIAA00&SELECT=S.sh%7c&R=35&Process+Action=display) |  |
|  | 20 |  limit 19 to english language [Limit not valid in Journals@Ovid; records were retained] | 5421 | Advanced | [Display Results](https://ovidsp-dc2-ovid-com.simsrad.net.ocs.mq.edu.au/ovid-b/ovidweb.cgi?&S=OPBDFPHCDOEBHAEOIPNJJHLECAGIAA00&SELECT=S.sh%7c&R=20&Process+Action=display)  [More](https://ovidsp-dc2-ovid-com.simsrad.net.ocs.mq.edu.au/ovid-b/ovidweb.cgi?&S=OPBDFPHCDOEBHAEOIPNJJHLECAGIAA00&SELECT=S.sh%7c&R=35&Process+Action=display) |  |
|  | 19 |  16 and 18 | 5741 | Advanced | [Display Results](https://ovidsp-dc2-ovid-com.simsrad.net.ocs.mq.edu.au/ovid-b/ovidweb.cgi?&S=OPBDFPHCDOEBHAEOIPNJJHLECAGIAA00&SELECT=S.sh%7c&R=19&Process+Action=display)  [More](https://ovidsp-dc2-ovid-com.simsrad.net.ocs.mq.edu.au/ovid-b/ovidweb.cgi?&S=OPBDFPHCDOEBHAEOIPNJJHLECAGIAA00&SELECT=S.sh%7c&R=35&Process+Action=display) |  |
|  | 18 |  ((Social support or Interpersonal relation* or Psychology, social or Social identity or Social identities or Sense of belonging or Social isolation or Loneliness or Unwanted or Alone or Social network* or Social interaction* or Social relationships or Social alienation or Social distance or Social connectivity or Social environment) and (intervention or Community intervention or Community-based intervention or Community based intervention or Group-based intervention or Group based intervention or Implementation or Social planning or Psychosocial intervention or Reminiscence therapy or Peer support intervention or Guideline* or Intervention stud* or Evaluation or Multi-component or Community based intervention or Multi-domain or Multicomponent or multi domain or Social activity or Social activities or community activities or social health or social health prevention)).mp. and (ageing or aging or older adults or older persons or elderly or aged or older or elder or geriatric or senior).m_titl. [mp=ti, ab, tx, ct, sh, hw, tn, ot, dm, mf, dv, kf, fx, dq, bt, nm, ox, px, rx, an, ui, sy, tc, id, tm] | 17185 | Advanced | [Display Results](https://ovidsp-dc2-ovid-com.simsrad.net.ocs.mq.edu.au/ovid-b/ovidweb.cgi?&S=OPBDFPHCDOEBHAEOIPNJJHLECAGIAA00&SELECT=S.sh%7c&R=18&Process+Action=display)  [More](https://ovidsp-dc2-ovid-com.simsrad.net.ocs.mq.edu.au/ovid-b/ovidweb.cgi?&S=OPBDFPHCDOEBHAEOIPNJJHLECAGIAA00&SELECT=S.sh%7c&R=35&Process+Action=display) |  |
|  | 17 |  15 and 16 | 47652 | Advanced | [Display Results](https://ovidsp-dc2-ovid-com.simsrad.net.ocs.mq.edu.au/ovid-b/ovidweb.cgi?&S=OPBDFPHCDOEBHAEOIPNJJHLECAGIAA00&SELECT=S.sh%7c&R=17&Process+Action=display)  [More](https://ovidsp-dc2-ovid-com.simsrad.net.ocs.mq.edu.au/ovid-b/ovidweb.cgi?&S=OPBDFPHCDOEBHAEOIPNJJHLECAGIAA00&SELECT=S.sh%7c&R=35&Process+Action=display) |  |
|  | 16 |  (cognition or memory* or brain or cognitive or cognitive).af. | 7833535 | Advanced | [Display Results](https://ovidsp-dc2-ovid-com.simsrad.net.ocs.mq.edu.au/ovid-b/ovidweb.cgi?&S=OPBDFPHCDOEBHAEOIPNJJHLECAGIAA00&SELECT=S.sh%7c&R=16&Process+Action=display)  [More](https://ovidsp-dc2-ovid-com.simsrad.net.ocs.mq.edu.au/ovid-b/ovidweb.cgi?&S=OPBDFPHCDOEBHAEOIPNJJHLECAGIAA00&SELECT=S.sh%7c&R=35&Process+Action=display) |  |
|  | 15 |  limit 14 to (elderly or elderly - focussed or "humans only (removes records about animals)") [Limit not valid in Journals@Ovid,APA PsycInfo; records were retained] | 79155 | Advanced | [Display Results](https://ovidsp-dc2-ovid-com.simsrad.net.ocs.mq.edu.au/ovid-b/ovidweb.cgi?&S=OPBDFPHCDOEBHAEOIPNJJHLECAGIAA00&SELECT=S.sh%7c&R=15&Process+Action=display)  [More](https://ovidsp-dc2-ovid-com.simsrad.net.ocs.mq.edu.au/ovid-b/ovidweb.cgi?&S=OPBDFPHCDOEBHAEOIPNJJHLECAGIAA00&SELECT=S.sh%7c&R=35&Process+Action=display) |  |
|  | 14 |  limit 13 to journal [Limit not valid in Journals@Ovid,Ovid MEDLINE(R),Ovid MEDLINE(R) Daily Update,Ovid MEDLINE(R) PubMed not MEDLINE,Ovid MEDLINE(R) In-Process,Ovid MEDLINE(R) Publisher; records were retained] | 79155 | Advanced | [Display Results](https://ovidsp-dc2-ovid-com.simsrad.net.ocs.mq.edu.au/ovid-b/ovidweb.cgi?&S=OPBDFPHCDOEBHAEOIPNJJHLECAGIAA00&SELECT=S.sh%7c&R=14&Process+Action=display)  [More](https://ovidsp-dc2-ovid-com.simsrad.net.ocs.mq.edu.au/ovid-b/ovidweb.cgi?&S=OPBDFPHCDOEBHAEOIPNJJHLECAGIAA00&SELECT=S.sh%7c&R=35&Process+Action=display) |  |
|  | 13 |  limit 12 to (embase or medline) [Limit not valid in Journals@Ovid,Ovid MEDLINE(R),Ovid MEDLINE(R) Daily Update,Ovid MEDLINE(R) PubMed not MEDLINE,Ovid MEDLINE(R) In-Process,Ovid MEDLINE(R) Publisher,APA PsycInfo; records were retained] | 80654 | Advanced | [Display Results](https://ovidsp-dc2-ovid-com.simsrad.net.ocs.mq.edu.au/ovid-b/ovidweb.cgi?&S=OPBDFPHCDOEBHAEOIPNJJHLECAGIAA00&SELECT=S.sh%7c&R=13&Process+Action=display)  [More](https://ovidsp-dc2-ovid-com.simsrad.net.ocs.mq.edu.au/ovid-b/ovidweb.cgi?&S=OPBDFPHCDOEBHAEOIPNJJHLECAGIAA00&SELECT=S.sh%7c&R=35&Process+Action=display) |  |
|  | 12 |  limit 11 to human [Limit not valid in Journals@Ovid; records were retained] | 81579 | Advanced | [Display Results](https://ovidsp-dc2-ovid-com.simsrad.net.ocs.mq.edu.au/ovid-b/ovidweb.cgi?&S=OPBDFPHCDOEBHAEOIPNJJHLECAGIAA00&SELECT=S.sh%7c&R=12&Process+Action=display)  [More](https://ovidsp-dc2-ovid-com.simsrad.net.ocs.mq.edu.au/ovid-b/ovidweb.cgi?&S=OPBDFPHCDOEBHAEOIPNJJHLECAGIAA00&SELECT=S.sh%7c&R=35&Process+Action=display) |  |
|  | 11 |  limit 10 to english | 81626 | Advanced | [Display Results](https://ovidsp-dc2-ovid-com.simsrad.net.ocs.mq.edu.au/ovid-b/ovidweb.cgi?&S=OPBDFPHCDOEBHAEOIPNJJHLECAGIAA00&SELECT=S.sh%7c&R=11&Process+Action=display)  [More](https://ovidsp-dc2-ovid-com.simsrad.net.ocs.mq.edu.au/ovid-b/ovidweb.cgi?&S=OPBDFPHCDOEBHAEOIPNJJHLECAGIAA00&SELECT=S.sh%7c&R=35&Process+Action=display) |  |
|  | 10 |  limit 9 to humans [Limit not valid in Journals@Ovid,APA PsycInfo; records were retained] | 81643 | Advanced | [Display Results](https://ovidsp-dc2-ovid-com.simsrad.net.ocs.mq.edu.au/ovid-b/ovidweb.cgi?&S=OPBDFPHCDOEBHAEOIPNJJHLECAGIAA00&SELECT=S.sh%7c&R=10&Process+Action=display)  [More](https://ovidsp-dc2-ovid-com.simsrad.net.ocs.mq.edu.au/ovid-b/ovidweb.cgi?&S=OPBDFPHCDOEBHAEOIPNJJHLECAGIAA00&SELECT=S.sh%7c&R=35&Process+Action=display) |  |
|  | 9 |  limit 8 to aged <65+ years> [Limit not valid in Journals@Ovid,Ovid MEDLINE(R),Ovid MEDLINE(R) Daily Update,Ovid MEDLINE(R) PubMed not MEDLINE,Ovid MEDLINE(R) In-Process,Ovid MEDLINE(R) Publisher; records were retained] | 81665 | Advanced | [Display Results](https://ovidsp-dc2-ovid-com.simsrad.net.ocs.mq.edu.au/ovid-b/ovidweb.cgi?&S=OPBDFPHCDOEBHAEOIPNJJHLECAGIAA00&SELECT=S.sh%7c&R=9&Process+Action=display)  [More](https://ovidsp-dc2-ovid-com.simsrad.net.ocs.mq.edu.au/ovid-b/ovidweb.cgi?&S=OPBDFPHCDOEBHAEOIPNJJHLECAGIAA00&SELECT=S.sh%7c&R=35&Process+Action=display) |  |
|  | 8 |  limit 7 to english language [Limit not valid in Journals@Ovid; records were retained] | 86900 | Advanced | [Display Results](https://ovidsp-dc2-ovid-com.simsrad.net.ocs.mq.edu.au/ovid-b/ovidweb.cgi?&S=OPBDFPHCDOEBHAEOIPNJJHLECAGIAA00&SELECT=S.sh%7c&R=8&Process+Action=display)  [More](https://ovidsp-dc2-ovid-com.simsrad.net.ocs.mq.edu.au/ovid-b/ovidweb.cgi?&S=OPBDFPHCDOEBHAEOIPNJJHLECAGIAA00&SELECT=S.sh%7c&R=35&Process+Action=display) |  |
|  | 7 |  limit 6 to (clinical trial or randomized controlled trial or controlled clinical trial or multicenter study) [Limit not valid in Journals@Ovid,APA PsycInfo; records were retained] | 88093 | Advanced | [Display Results](https://ovidsp-dc2-ovid-com.simsrad.net.ocs.mq.edu.au/ovid-b/ovidweb.cgi?&S=OPBDFPHCDOEBHAEOIPNJJHLECAGIAA00&SELECT=S.sh%7c&R=7&Process+Action=display)  [More](https://ovidsp-dc2-ovid-com.simsrad.net.ocs.mq.edu.au/ovid-b/ovidweb.cgi?&S=OPBDFPHCDOEBHAEOIPNJJHLECAGIAA00&SELECT=S.sh%7c&R=35&Process+Action=display) |  |
|  | 6 |  limit 5 to ("all aged (65 and over)" or "aged (80 and over)" or "380 aged <age 65 yrs and older>" or "390 very old <age 85 yrs and older>") [Limit not valid in Journals@Ovid,Embase,Ovid MEDLINE(R),Ovid MEDLINE(R) Daily Update,Ovid MEDLINE(R) PubMed not MEDLINE,Ovid MEDLINE(R) In-Process,Ovid MEDLINE(R) Publisher,APA PsycInfo; records were retained] | 159142 | Advanced | [Display Results](https://ovidsp-dc2-ovid-com.simsrad.net.ocs.mq.edu.au/ovid-b/ovidweb.cgi?&S=OPBDFPHCDOEBHAEOIPNJJHLECAGIAA00&SELECT=S.sh%7c&R=6&Process+Action=display)  [More](https://ovidsp-dc2-ovid-com.simsrad.net.ocs.mq.edu.au/ovid-b/ovidweb.cgi?&S=OPBDFPHCDOEBHAEOIPNJJHLECAGIAA00&SELECT=S.sh%7c&R=35&Process+Action=display) |  |
|  | 5 |  ((Social support or Interpersonal relation* or Psychology, social or Social identity or Social identities or Sense of belonging or Social isolation or Loneliness or Unwanted or Alone or Social network* or Social interaction* or Social relationships or Social alienation or Social distance or Social connectivity or Social environment) and (intervention or Community intervention or Community-based intervention or Community based intervention or Group-based intervention or Group based intervention or Implementation or Social planning or Psychosocial intervention or Reminiscence therapy or Peer support intervention or Guideline* or Intervention stud* or Evaluation or Multi-component or Community based intervention or Multi-domain or Multicomponent or multi domain or Social activity or Social activities or community activities or social health or social health prevention) and (ageing or aging or older adults or older persons or elderly or aged or older or elder or geriatric or senior)).af. | 297385 | Advanced | [Display Results](https://ovidsp-dc2-ovid-com.simsrad.net.ocs.mq.edu.au/ovid-b/ovidweb.cgi?&S=OPBDFPHCDOEBHAEOIPNJJHLECAGIAA00&SELECT=S.sh%7c&R=5&Process+Action=display)  [More](https://ovidsp-dc2-ovid-com.simsrad.net.ocs.mq.edu.au/ovid-b/ovidweb.cgi?&S=OPBDFPHCDOEBHAEOIPNJJHLECAGIAA00&SELECT=S.sh%7c&R=35&Process+Action=display) |  |
|  | 4 |  limit 3 to humans [Limit not valid in Journals@Ovid,APA PsycInfo; records were retained] | 138237 | Advanced | [Display Results](https://ovidsp-dc2-ovid-com.simsrad.net.ocs.mq.edu.au/ovid-b/ovidweb.cgi?&S=OPBDFPHCDOEBHAEOIPNJJHLECAGIAA00&SELECT=S.sh%7c&R=4&Process+Action=display)  [More](https://ovidsp-dc2-ovid-com.simsrad.net.ocs.mq.edu.au/ovid-b/ovidweb.cgi?&S=OPBDFPHCDOEBHAEOIPNJJHLECAGIAA00&SELECT=S.sh%7c&R=35&Process+Action=display) |  |
|  | 3 |  limit 2 to human [Limit not valid in Journals@Ovid; records were retained] | 138237 | Advanced | [Display Results](https://ovidsp-dc2-ovid-com.simsrad.net.ocs.mq.edu.au/ovid-b/ovidweb.cgi?&S=OPBDFPHCDOEBHAEOIPNJJHLECAGIAA00&SELECT=S.sh%7c&R=3&Process+Action=display)  [More](https://ovidsp-dc2-ovid-com.simsrad.net.ocs.mq.edu.au/ovid-b/ovidweb.cgi?&S=OPBDFPHCDOEBHAEOIPNJJHLECAGIAA00&SELECT=S.sh%7c&R=35&Process+Action=display) |  |
|  | 2 |  limit 1 to english language [Limit not valid in Journals@Ovid; records were retained] | 141964 | Advanced | [Display Results](https://ovidsp-dc2-ovid-com.simsrad.net.ocs.mq.edu.au/ovid-b/ovidweb.cgi?&S=OPBDFPHCDOEBHAEOIPNJJHLECAGIAA00&SELECT=S.sh%7c&R=2&Process+Action=display)  [More](https://ovidsp-dc2-ovid-com.simsrad.net.ocs.mq.edu.au/ovid-b/ovidweb.cgi?&S=OPBDFPHCDOEBHAEOIPNJJHLECAGIAA00&SELECT=S.sh%7c&R=35&Process+Action=display) |  |
|  | 1 |  ((Social support or Interpersonal relation* or Psychology, social or Social identity or Social identities or Sense of belonging or Social isolation or Loneliness or Unwanted or Alone or Social network* or Social interaction* or Social relationships or Social alienation or Social distance or Social connectivity or Social environment) and (intervention or Community intervention or Community-based intervention or Community based intervention or Group-based intervention or Group based intervention or Implementation or Social planning or Psychosocial intervention or Reminiscence therapy or Peer support intervention or Guideline* or Intervention stud* or Evaluation or Multi-component or Community based intervention or Multi-domain or Multicomponent or multi domain or Social activity or Social activities or community activities or social health or social health prevention) and (ageing or aging or older adults or older persons or elderly or aged or older or elder or geriatric or senior)).mp. [mp=ti, ab, tx, ct, sh, hw, tn, ot, dm, mf, dv, kf, fx, dq, bt, nm, ox, px, rx, an, ui, sy, tc, id, tm] | 147920 | Advanced | [Display Results](https://ovidsp-dc2-ovid-com.simsrad.net.ocs.mq.edu.au/ovid-b/ovidweb.cgi?&S=OPBDFPHCDOEBHAEOIPNJJHLECAGIAA00&SELECT=S.sh%7c&R=1&Process+Action=display)  [More](https://ovidsp-dc2-ovid-com.simsrad.net.ocs.mq.edu.au/ovid-b/ovidweb.cgi?&S=OPBDFPHCDOEBHAEOIPNJJHLECAGIAA00&SELECT=S.sh%7c&R=35&Process+Action=display) |  |

**Supplementary Table 2. Behaviour change techniques identified in the studies**

| **Article** | **BCT** | **Example** |
| --- | --- | --- |
| **Ahessey 2016** | Goal setting (behaviour) | “After verbal and written consent was obtained, participants were randomly assigned to two groups; a treatment (choir) group (n=20) who would actively participate in the choir for 12 weeks and a control group (n=20) who would receive standard nursing care. The control group were informed that they would receive four choral sessions once the study had terminated.” |
|  | Action planning | “The choir group attended the therapy session weekly on Friday mornings for approximately 1 hour.”  “The participants were required to attend a minimum of 8 out of the 12 sessions to be included in the study.” |
|  | Instruction on how to perform a behaviour | “Each choir session was facilitated by a qualified music therapist who led all the singing and accompanied on a digital piano.”  “The principles of adult social learning in which mistakes are ignored and efforts are noted and encouraged, the ‘no mistake approach’ were used in the sessions…” |
|  | Monitoring of emotional consequences | Participants were encouraged to select their preferred songs from the list of songs compiled from a previous music therapy choir the researcher had been involved with. |
| **Akanuma 2010** | Goal setting (behaviour) | “We hypothesized that the frontal lobe associated with social interaction was being stimulated. To test this hypothesis, we studied 24 patients with vascular dementia. In addition to conventional care, a 1-h session of GRA with RO was provided once a week for 3 months in the GRA-RO arm (n = 12).” |
|  | Action planning | “In addition to conventional care, a 1-h session of GRA and RO was provided by an author (K.A.) with the staff once a week for 3 months to the patients in the GRA-RO arm. The sessions were held in a separate room from their daily activities.”  “Briefly, after greetings (2 min) and the RO (time and place) with confirmation of participants' attendance (3 min), the GRA (50 min) was performed by talking about topics related to participants' past…” |
|  | Instruction on how to perform a behaviour | The participants in the GRA-RO arm were prompted to talk about their past with the staff and then sung a song to close off section. |
| **Dodge 2015** | Goal setting (behaviour) | “Daily 30-minute face-to-face communications were conducted during a 6-week trial period in the intervention group. The control group received only a weekly telephone interview.”  “The intervention group engaged in face-to-face conversations with trained interviewers 5 days a week (Monday through Friday) for 6 weeks by way of a dedicated video chat-enabled PC provided to each subject. Each conversational session was designed to last 30 to 35 minutes. The control group received weekly telephone calls to assess their social engagement activities during the previous week (i.e., no PC or Internet provided).” |
|  | Instruction on how to perform a behaviour | The intervention group was led by trained interviewers in a dedicated video chat enabled PC provided. |
|  | Adding objects to the environment | “We created our own version of a chat system in which participants did not need to know how to use a computer, other than to touch the touch screen of a computer preconfigured to receive calls and automatically begin the conversational session. The study computer was enabled to record the trial sessions and store encrypted audio data automatically. Technical support personnel visited each participant’s home and set up the equipment. ” |
| **Gudex 2010** | Goal setting (behaviour) | “Nursing home staff in the Intervention Group attended a standardised course in reminiscence (two whole and two half-days over a 9-month period) that comprised formal teaching, group work and discussion sessions (conducted by MK). During the intervention period (August 2006 - August 2007) nursing staff were expected to use three forms of reminiscence: general reminiscence…specific reminiscence…spontaneous reminiscence” |
|  | Action planning | “General reminiscence: group sessions run by 1-2 nurses for typically 2-8 residents with similar back- grounds or interests; structured around a chosen theme, often based on a reminiscence box containing a variety of tools, photos, books, music etc. and involving senses of sight, touch, smell, hearing.  • Specific reminiscence: sessions for 1-2 residents structured around a theme and tailored to the individual resident's communication needs; often using family photos, reminiscence games and activities e.g. baking.  • Spontaneous reminiscence: informal use of comments during regular daily activities (e.g. dressing, mealtimes, preparing for bed) to elicit a resident's memories of earlier life experiences; can start as an individual contact that spreads to involve others sitting nearby” |
|  | Social support (unspecified) | “A reminiscence trainer (AM) visited each nursing home 5-8 times during the intervention period to provide guidance and encouragement in the use of reminiscence material and activities. ” |
|  | Instruction on how to perform a behaviour | “Nursing home staff in the Intervention Group attended a standardised course in reminiscence that comprised formal teaching, group work and discussion sessions…” |
|  | Monitoring of emotional consequences | “At the first baseline data collection, nursing staff completed the questionnaires on residents' agitated behaviour, quality of life and general level of functioning together with a project interviewer. Hereafter the nursing staff completed the questionnaires themselves. ” |
|  | Demonstration of the behavior | “During the intervention period (August 2006 - August 2007) nursing staff were expected to use three forms of reminiscence: general…specific…spontaneous” |
|  | Credible source | “Nursing home staff in the Intervention Group attended a standardised course in reminiscence that comprised formal teaching, group work and discussion sessions…” and “The reminiscence approach used in the current study builds further on the approach used by the Danish Centre for Reminiscence (Noorebro Erindringscentre). Greater emphasis has been given to categorising reminiscence as general, specific or spontaneous…” |
|  | Restructuring the physical environment | “Thirty reminiscence boxes were circulated between the five IG nursing homes during the intervention period. These boxes could also be used to make small exhibitions, which could be visited by staff, residents and visitors…” |
|  | Adding objects to the environment | “Thirty reminiscence boxes were circulated between the five IG nursing homes during the intervention period. These boxes could also be used to make small exhibitions, which could be visited by staff, residents and visitors…” |
| **Iizuka 2018** | Goal setting (behaviour) | “Twelve 1‐hour Go classes were held once a week in a community centre. Each class involved attending a lecture on basic Go rules and techniques (15 minutes), solving Go exercises (10 minutes), learning tactics using a model game called kifu‐narabe (10 minutes), and playing Go with others (two to four participants) (25 minutes).”  “We provided each participant with a tablet computer during the intervention period. Using the tablet, each participant in the NFG individually attended the same Go classes as those in the FG for the same time and period and at the same frequency.”  “Participants attended a 2‐hour lecture on health maintenance, includ- ing topics such as frailty, sarcopenia, and depression, once a month. ” |
|  | Action planning | “Twelve 1‐hour Go classes were held once a week in a community centre. Each class involved attending a lecture on basic Go rules and techniques (15 minutes), solving Go exercises (10 minutes), learning tactics using a model game called kifu‐narabe (10 minutes), and playing Go with others (two to four participants) (25 minutes).”  “We provided each participant with a tablet computer during the intervention period. Using the tablet, each participant in the NFG individually attended the same Go classes as those in the FG for the same time and period and at the same frequency.”  “Participants attended a 2‐hour lecture on health maintenance, includ- ing topics such as frailty, sarcopenia, and depression, once a month.” |
|  | Instruction on how to perform a behaviour | Go instructors were there to instruct/teach the participants the game of Go. |
|  | Demonstration of the behavior | “Each class involved attending a lecture on basic Go rules and techniques (15 minutes), solving Go exercises (10 minutes), learning tactics using a model game called kifu‐narabe (10 minutes), and playing Go with others (two to four participants) (25 minutes).” |
|  | Generalisation of a target behavior | “Between classes, the participants were required to complete homework assignments (one per day for 6 days) that consisted of Go exercises created by the instructors.” |
|  | Credible source | Go instructors and researchers were involved in the study. |
|  | Adding objects to the environment | Tablet computers were provided to the non face to face Go group. |
| **Melendez-Moral 2013** | Goal setting (behaviour) | “The present study’s purpose was to research the usefulness of reminiscence intervention in a sample of institutionalized, elderly adults. The intervention was conducted as a group, lasted eight sessions, and included a control group for comparison using a single-blind design. Its central objective was to improve participants’ depressive symptomatology, self-esteem, life satisfaction, and psychological well-being.” |
|  | Action planning | “Group sessions were conducted by a psychologist, who directed the entire intervention. Eight 60-minute sessions were held, each with a similar structure.” |
|  | Instruction on how to perform a behaviour | “Activities followed and each session ended with an evaluation of the session itself, and with comments about the next session’s main points. Each session was geared toward a specific theme and…spontaneously and deliberately introduced triggers, all the while utilizing an interpersonal style in which people share their memories with others through storytelling. The sessions themselves each focused on a specific topic: from childhood through old age; remembering where I’ve lived: my town/city; games from childhood and youth; popular songs; holidays and special days; the movies over time; and remembering my grandmother, which spanned two sessions.” |
|  | Monitoring of emotional consequences | “The sessions themselves each focused on a specific topic: from childhood through old age; remembering where I’ve lived: my town/city; games from childhood and youth; popular songs; holidays and special days; the movies over time; and remembering my grandmother, which spanned two sessions.” |
|  | Verbal persuasion about capability | “Activities followed and each session ended with an evaluation of the session itself, and with comments about the next session’s main points. Each session was geared toward a specific theme and…spontaneously and deliberately introduced triggers, all the while utilizing an interpersonal style in which people share their memories with others through storytelling. |
| **Mortimer 2012** | Goal setting (behaviour) | “Participants assigned to this group met with a Tai Chi master and assistant three times per week in the morning in Jing An Park or at a nearby gymnasium depending on weather conditions. Each session included 20 min of warm-up exercises (lower back and hamstring stretching, gentle calisthenics, and balance training), 20 min of Tai Chi practice, and 10 min of cool-down exercises.  Participants assigned to this group met with two group leaders three times per week in the morning in Jing An Park and were encouraged to walk quickly around a 400 meter circular route. Each session consisted of 10 min of warm-up stretching, 30 min of brisk walking, and 10 min of cool-down exercises.  Participants assigned to this group met with a group leader and an assistant for 1 h three times a week in the morning at the neighborhood community center.” |
|  | Action planning | “Participants assigned to this group met with a Tai Chi master and assistant three times per week in the morning in Jing An Park or at a nearby gymnasium depending on weather conditions. Each session included 20 min of warm-up exercises (lower back and hamstring stretching, gentle calisthenics, and balance training), 20 min of Tai Chi practice, and 10 min of cool-down exercises.  Participants assigned to this group met with two group leaders three times per week in the morning in Jing An Park and were encouraged to walk quickly around a 400 meter circular route. Each session consisted of 10 min of warm-up stretching, 30 min of brisk walking, and 10 min of cool-down exercises.  Participants assigned to this group met with a group leader and an assistant for 1 h three times a week in the morning at the neighborhood community center.” |
|  | Feedback on behaviour | “They were asked to take 100 steps and the sensitivity and positioning of the pedometer was adjusted to assure accurate measurement.” |
|  | Self-monitoring of behaviour | “At the termination of each session, the pedometers were collected and the number of steps taken by each participant at that session recorded. A record of the number of steps taken for every participant at each session over the 40 week period was maintained.” |
|  | Biofeedback | “At the termination of each session, the pedometers were collected and the number of steps taken by each participant at that session recorded. A record of the number of steps taken for every participant at each session over the 40 week period was maintained.” |
|  | Instruction on how to perform a behaviour | There were directions given from the group leader and an assistant for the participants assigned to the social interaction group. |
|  | Demonstration of the behavior | The different intervention groups were led by their respective leaders and performed accordingly. |
|  | Credible source | Tai Chi master, group leaders for walking and social intervention groups were mentioned. |
| **Nakatsuka 2015** | Goal setting (behaviour) | **“**Each cluster of 6–9 persons received one of the three interventions (CI, PA or GRA) that was assigned to the cluster. All of them consisted of 12 group sessions and 12 home assignments. **”** |
|  | Action planning | **“**Each group session was held once per week and contained an intervention program (60 min in total) that was interrupted by a tea break (5 min). Instructions about the tasks for the home assignment were given at the end of each group session (5 min). The home assignments were designed according to the intervention methods and contained tasks that require about 1 h to be completed. Written instructions were also given to the participants as an aid to successfully complete the home assignments.” |
|  | Review behaviour goal(s) | “The RPE is especially useful in practice or clinical settings because self-monitoring how hard one’s body is working may easily allow for self-adjustment of the intensity of activity. To assign the Borg RPE, the subjects selected one of the scores ranging from 6 to 20, where 6 and 20 correspond to ‘no exertion at all’ and ‘maximum exertion’, respectively.” |
|  | Feedback on behaviour | **“**To assign the Borg RPE, the subjects selected one of the scores ranging from 6 to 20, where 6 and 20 correspond to ‘no exertion at all’ and ‘maximum exertion’, respectively.**”** |
|  | Self-monitoring of behaviour | “The RPE is especially useful in practice or clinical settings because self-monitoring how hard one’s body is working may easily allow for self-adjustment of the intensity of activity. To assign the Borg RPE, the subjects selected one of the scores ranging from 6 to 20, where 6 and 20 correspond to ‘no exertion at all’ and ‘maximum exertion’, respectively.” |
|  | Biofeedback | **“**Each session and home assignment consisted of 6 sets of exercises of 3–5 min of duration that were done every 10 min. The exercises were composed of walking and step aerobics using STEPWELL 2**”** |
|  | Instruction on how to perform a behaviour | The participants were instructed on how to perform by their instructors for each respective intervention group. |
|  | Monitoring of emotional consequences | **“**For the GRA, each session and home assignment consisted of talks about the current days and events of daily life (reality orientation) and discussions about memories of earlier days (reminiscence approach).” |
|  | Demonstration of the behavior | **“**Instructions about the tasks for the home assignment were given at the end of each group session….Written instructions were also given to the participants as an aid to successfully complete the home assignments.” |
|  | Generalisation of a target behavior | **“T**he RPE is a scale to document the subjective exertion, and it may be used for prescribing and monitoring exercise intensity. Although this is a subjective measure, a person’s exertion rating may provide a fairly good estimate of biological parameters including the actual heart rate.**”** |
|  | Credible source | One instructor and two assistants were assigned for each intervention group. |
|  | Adding objects to the environment | Crossword puzzles, maze solving, card games such as the ‘Old Maid’, sudoku |
| **Park 2014** | Goal setting (behaviour) | **“**Participants assigned to the productive-engagement conditions were directed to spend an average of 15 hr per week in the Synapse environment: 5 hr of formal instruction and 10 hr completing course assignments. Participants received instruction in groups of six.**”** |
|  | Action planning | **“**Participants were instructed by a professional photographer who trained them to use cameras and develop computer skills required to use professional photography software for photo editing.**”**  **“**The quilt condition had the same format as the photo condition and was under the direction of a professional quilting instructor. All participants learned basic skills and progressed to complete complex, individual projects using computer-driven sewing machines.**”**  “The social condition mimicked a social club: It involved instructor- directed activities, such as cooking, playing games, watching movies, reminiscing, and going on regular field trips organized around a different topic, such as travel or history, each week. The social-group curriculum relied as much as possible on participants' existing knowledge, with no formal knowledge acquisition.” |
|  | Instruction on how to perform a behaviour | **“**Participants were instructed by a professional photographer who trained them to use cameras and develop computer skills required to use professional photography software for photo editing.**”** |
|  | Demonstration of the behavior | Both the photo and quilt condition were instructed and trained by their respective professionals. |
|  | Credible source | Professional photographer, professional quilting instructor |
|  | Adding objects to the environment | Cameras, quilts, movies, games, field trips |
| **Pena 2014** | Goal setting (behaviour) | **“**REHACOP is a structured program using paper-pencil tasks29 (based on restoration, compensation, and optimization strategies of rehabilitation) with a gradual level of cognitive effort and demand. REHACOP trains different cognitive domains, such as attention, memory, processing speed, language, executive functioning, and social cognition.**”** |
|  | Action planning | **“**REHACOP group remediation with patients with PD consisted of the following: attention unit (4 weeks) training sustained, selective, alternant, and divided attention; memory unit (3 weeks) focusing on visual and verbal learning, recall, and recognizing memory; language unit (3 weeks) including grammar, syntax, vocabulary, verbal fluency, verbal comprehension, and abstract language; executive functions unit (2 weeks) training cognitive planning, proverbs, and analogies; and social cognition unit (1 week) exercising TOM, social reasoning, and moral dilemmas. **”** |
|  | Instruction on how to perform a behaviour | **“**In this study, 2 psychologists conducted the REHACOP group attending 60-minute-long sessions 3 days per week at ASPARBI (2 groups) or the Hospital of Galdakao (1 group). Both psychologists prepared the sessions together, used the same materials and instructions, and received the same training on REHACOP.**”** |
| **Pitkala 2011** | Goal setting (behaviour) | “The intervention groups met at the intervention centers once a week for 3 months, thus, altogether 12 times. Each group consisted of seven to eight elderly participants and two professional group leaders. The group meetings usually lasted for 6 hours.”  “The professional group leaders aimed at enhancing security as well as equal and lively communication between participants. Maturation of the group cycle during the 3 months of group intervention helped the participants to experience the ups and downs of their newly developing friendships.” |
|  | Problem solving | “The idea of the intervention was that the participants of the group would share their experiences with others of their own age, discuss their feelings of loneliness, receive peer support from one another, dare to surpass their own limits, and develop feelings of solidarity.” |
|  | Action planning | “The intervention groups met at the intervention centers once a week for 3 months, thus, altogether 12 times. Each group consisted of seven to eight elderly participants and two professional group leaders. The group meetings usually lasted for 6 hours.” |
|  | Review behaviour goal(s) | “In the “therapeutic writing” groups, the participants wrote about their past, their loneliness, and their feelings about the group meetings. They shared their writings and experiences in discussions with their group members and reminisced about their pasts.” |
|  | Feedback on behaviour | “The principles of this socially stimulating intervention were same in all groups, irrespective of the group subactivity. The common training and tutor- ing of the group leaders ensured this. The professional group leaders aimed at enhancing security as well as equal and lively communication between participants.” |
|  | Social support (unspecified) | “By taking advantage of group dynamics and the normal maturation of a group cycle, the group leaders actively enhanced peer support, promoted friend- ships, and supported participants’ empowerment.” |
|  | Social support (emotional) | “By taking advantage of group dynamics and the normal maturation of a group cycle, the group leaders actively enhanced peer support, promoted friend- ships, and supported participants’ empowerment.” |
|  | Instruction on how to perform a behaviour | “By taking advantage of group dynamics and the normal maturation of a group cycle, the group leaders actively enhanced peer support, promoted friend- ships, and supported participants’ empowerment. They worked more as facilitators than as active leaders.” |
|  | Monitoring of emotional consequences | “The idea of the intervention was that the participants of the group would share their experiences with others of their own age, discuss their feelings of loneliness, receive peer support from one another, dare to surpass their own limits, and develop feelings of solidarity. This would in turn lead to their empowerment, better self-respect, and mastery over their own lives.” |
|  | Demonstration of the behavior | “The idea of the intervention was that the participants of the group would share their experiences with others of their own age, discuss their feelings of loneliness, receive peer support from one another, dare to surpass their own limits, and develop feelings of solidarity. This would in turn lead to their empowerment, better self-respect, and mastery over their own lives.” |
|  | Credible source | Study nurses, registered nurses, occupational therapists, physiotherapists |
|  | Reduce negative emotions | “As the group matured and the relationships became stronger, the participants also dared to be critical of each other and the group leaders. Over time, successfully solved group problems led to an even stronger cohesion between group members and their mutual responsibility for the group. ” |
|  | Restructuring the physical environment | The participants in the intervention group met at intervention centers. |
|  | Valued self-identity | “This would in turn lead to their empowerment, better self-respect, and mastery over their own lives.” |
|  | Verbal persuasion about capability | “This would in turn lead to their empowerment, better self-respect, and mastery over their own lives.” |
| **Tanaka 2012** | Goal setting (behaviour) | **“**The shape, voice, and motion features of the communication robot resemble those of a 3-year-old boy, while the control robot was not designed to talk or nod. Before living with the robot and 4 and 8 weeks after living with the robot, experiments were conducted to evaluate a variety of cognitive functions as well as saliva cortisol, sleep, and subjective fatigue, motivation, and healing.**”** |
|  | Problem solving | **“**While living with the robot, subjects refrained from strenuous mental and physical activity and followed normal dietary behaviour, drinking patterns, and sleeping hours, and completed questionnaires dealing with diet, sleep, activity, physical and mental condition, life events, and communication with the robot every day at home.” |
|  | Action planning | **“…**the participants were randomly assigned to 2 groups, matched for age and MMSE score, to  live with either a communication robot or a control robot at home for 8 weeks. Experiments were conducted before (baseline) and 4 and 8 weeks after the start of living with the robot**”** |
|  | Instruction on how to perform a behaviour | **“**In this study, elderly female volunteers living alone were randomized to living  with either the communication robot or the control robot at home for 8 weeks.**”** |
|  | Monitoring of emotional consequences | **“**While living with the robot, subjects refrained from strenuous mental and physical activity and followed normal dietary behaviour, drinking patterns, and sleeping hours, and completed questionnaires dealing with diet, sleep, activity, physical and mental condition, life events, and communication with the robot every day at home.” |
|  | Adding objects to the environment | Live in robots |
| **Tesky 2011** | Goal setting (behaviour) | **“**AKTIVA program was a randomized controlled training study, using a 3-group de- sign – two intervention groups and a control group. Participants were randomly assigned to either an intervention group or the control group. Participants from both intervention groups received training in the AKTIVA program. Intervention Group 2 received additional nutritional education and physical exercise program to determine whether the combination of AKTIVA training along with nutritional and exercise counseling was more effective on outcome measures than the AKTIVA program alone.” |
|  | Action planning | **“**Participants were randomly assigned to either an intervention group or the control group. Participants from both intervention groups received training in the AKTIVA program. Intervention Group 2 received additional nutritional education and physical exercise program to determine whether the combination of AKTIVA training along with nutritional and exercise counselling was more effective on outcome measures than the AKTIVA program alone. Participants in the second intervention group were required to receive a physical checkup and to take introductory courses in several sports and physical activities (e.g., gymnastics, walking, yoga) as well as a nutrition workshop, and to maintain a movement diary.**”** |
|  | Instruction on how to perform a behaviour | “In groups of 10–12 individuals, participants were informed about dementia, risk factors, and individual prevention strategies over the duration of eight weekly sessions. After educating participants about the above, we gave information to participants pertaining to age-related changes (i.e., cognitive decline, loss of sensory abilities, experience of life, freetime in retirement), coping strategies to combat the effects of aging, and goal development for a healthy, active, and dementia-pre- venting lifestyle. Additional information about the importance and principles of motivation, self-motivation, and self-awareness was part of the intervention sessions.” |
|  | Demonstration of the behavior | “In groups of 10–12 individuals, participants were informed about dementia, risk factors, and individual prevention strategies over the duration of eight weekly sessions. After educating participants about the above, we gave information to participants pertaining to age-related changes (i.e., cognitive decline, loss of sensory abilities, experience of life, freetime in retirement), coping strategies to combat the effects of aging, and goal development for a healthy, active, and dementia-pre- venting lifestyle. Additional information about the importance and principles of motivation, self-motivation, and self-awareness was part of the intervention sessions.” |
| **Vidovich 2015** | Goal setting (behaviour) | “Each program was administered to groups of six to nine individuals, with participants attending ten 90- minute sessions over a 5-week period (two sessions per week).” |
|  | Action planning | “Each program was administered to groups of six to nine individuals, with participants attending ten 90- minute sessions over a 5-week period (two sessions per week).” |
|  | Instruction on how to perform a behaviour | “The sessions and strategies incorporated elements of cognitive rehabilitation, cognitive stimulation, and cognitive training. This form of CFI was chosen to overcome some of the limitations of other approaches, offer a format suitable for group delivery, be person-centered, and to address the particular needs of people with MCI.” |
|  | Demonstration of the behavior | “A clinical neuropsychologist ran each of the groups, exposing the participants to the same intervention format, amount of instructor time, and social contact. All participants were given a manual containing the session content and material. ” |
| **Zimmerman 2014** | Goal setting (behaviour) | “Participants received 6 weeks sessions of WM training or poetry activities and each session lasting two hour.” |
|  | Action planning | “Participants received 6 weeks sessions of WM training or poetry activities and each session lasting two hour. The main goal of WM training program was to improve skills that require components of this construct described by (Baddeley et al., 2009), like phonological loop, visual-spatial sketchpad, episodic buffer, and central executive.”  “The program was composed by activities having as main goal to improve reading and interpretation abilities of poetry.” |
|  | Instruction on how to perform a behaviour | “More basic processing like phonological loop and visual-spatial sketchpad were strongly emphasized during the first sessions, while more demanding abilities, like episodic buffer and central executive, were trained in the latter meetings.”  “The program was composed by activities having as main goal to improve reading and interpretation abilities of poetry. Tasks consisted of reading of poems, listening to songs, visualization of pictures related to the poems and the songs, discussions about the subjective meaning, main ideas, personal feelings about data presented, presentation of such ideas in a group, interpretation and identification of metaphors used in the poems, production of metaphors and use of personal photography to the discussion of poems that address memories of their own life span.” |

NB: BCT=Behaviour Change Technique; CFI=cognitive-focused interventions; CI=cognitive interventions; GRA=group reminiscence approach; MCI=Mild Cognitive Impairment; MMSE=Mini-Mental State Examination; PA=physical activity; REHACOP=integrative cognitive training program; RO=reality orientation; TOM=theory of mind; WM=working memory.
